# Adsorption and Diffusion of CH_4_, N_2_, and Their Mixture in MIL-101(Cr): A Molecular Simulation Study

**DOI:** 10.1021/acs.jced.4c00233

**Published:** 2024-08-22

**Authors:** Yimin Shao, Shanshan Wang, Liangliang Huang, Shenghong Ju, Xianfeng Fan, Wei Li

**Affiliations:** † Institute for Materials and Processes, School of Engineering, The University of Edinburgh, Edinburgh EH9 3FB, Scotland, U.K.; ‡ College of Chemical Engineering, International Innovation Center for Forest Chemicals and Materials, Nanjing Forestry University, Nanjing, Jiangsu 210037, P.R. China; § School of Sustainable Chemical, Biological and Materials Engineering, University of Oklahoma, Norman, Oklahoma 73019, United States; ∥ China-UK Low Carbon College, Shanghai Jiao Tong University, Shanghai 201306, China

## Abstract

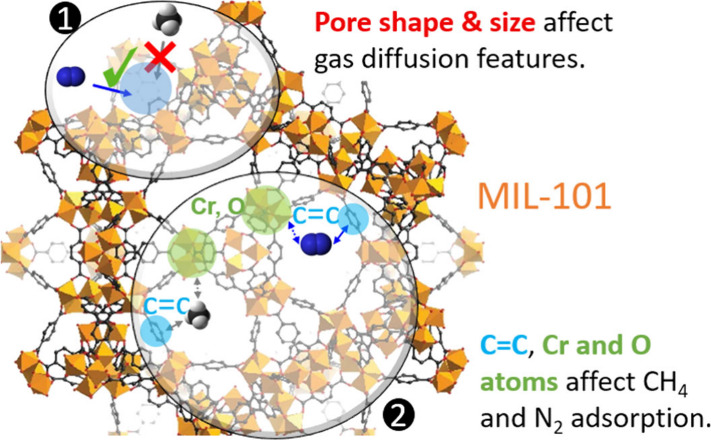

A comprehensive quantitative grasp of methane (CH_4_), nitrogen (N_2_), and their mixture’s adsorption and diffusion in MIL-101(Cr) is crucial for wide and important applications, e.g., natural gas upgrading and coal-mine methane capturing. Previous studies often overlook the impact of gas molecular configuration and MIL-101 topology structure on adsorption, lacking quantitative assessment of primary and secondary adsorption sites. Additionally, understanding gas mixture adsorption mechanisms remains a research gap. To bridge this gap and to provide new knowledge, we utilized Monte Carlo and molecular dynamics simulations for computing essential MIL-101 properties, encompassing adsorption isotherms, density profiles, self-diffusion coefficients, radial distribution function (RDF), and CH_4_/N_2_ selectivity. Several novel and distinctive findings are revealed by the atomic-level analysis, including (1) the significance of C=C double bond of the benzene ring within MIL-101 for CH_4_ and N_2_ adsorption, with Cr and O atoms also exerting notable effects. (2) Density distribution analysis reveals CH_4_’s preference for large and medium cages, while N_2_ is evenly distributed along pentagonal and triangular window edges and small tetrahedral cages. (3) Calculations of self-diffusion and diffusion activation energies suggest N_2_’s higher mobility within MIL-101 compared to CH_4_. (4) In the binary mixture, the existence of CH_4_ can decrease the diffusion coefficient of N_2_. In summary, this investigation provides valuable microscopic insights into the adsorption and diffusion phenomena occurring in MIL-101, thereby contributing to a comprehensive understanding of its potential for applications, e.g., natural gas upgrading and selective capture of coal-mine methane.

## 1 Introduction

Global warming, resulting from the escalating levels of greenhouse gases, represents an undeniable and urgent challenge in the twenty-first century.^1^ Methane (CH_4_), one of the six primary greenhouse gases regulated by the Kyoto Protocol, possesses a warming potential over 20 times greater than that of carbon dioxide (CO_2_).^2^ Annual methane emissions from underground coal mines have exhibited a range from 2.03 to 2.87 billion cubic meters in recent decades.^3^ Projections by the US Environmental Protection Agency forecast that global methane emissions from coal mines will surpass 784.3 MtCO2e by 2030.^4^ Furthermore, coal-mine methane, with its low CH_4_ content (50%), is unsuitable for direct utilization as chemicals or fuels.^5^ Consequently, the selective separation of CH_4_ from N_2_ in coal-mine methane holds immense significance and vitality. This separation process has the potential to not only yield valuable chemicals and fuels, leading to economic benefits, but also contribute to the reduction of atmospheric greenhouse gas emissions, thereby aiding in the fight against global warming.^6^

The cryogenic distillation process, which relies on the difference in boiling temperatures between CH_4_ (112 K) and N_2_ (77 K) to separate CH_4_ from N_2_ in coal-mine methane, is the most widely employed in industry.^7^ However, this approach is deemed economically impractical, energy-intensive, and environmentally unfavorable for coal-mine methane capture. This is primarily due to the low CH_4_ content in coal-mine methane and the demanding operating conditions, such as low temperatures and high pressures, required for cryogenic distillation.^8^ An alternative approach that has gained significant attention is adsorption separation, which employs porous materials and operates at ambient temperature and pressure. This method is widely recognized as an energy-efficient and cost-effective solution for capturing low-concentration CH_4_ from N_2_ in coal-mine methane.^9^

Extensive research has been conducted on conventional porous materials, including activated carbons,^10^ zeolites,^11^ and molecular sieves,^12^ to evaluate their effectiveness in separating CH_4_/N_2_ mixtures. However, the challenge of achieving a high CH_4_/N_2_ selectivity remains unresolved. To address this issue, it is imperative to explore novel types of porous materials. Metal–organic frameworks (MOFs) have emerged as promising candidates for selective gas separations due to their tunable pore size and chemistry.^13^ Several MOFs have been investigated for CH_4_/N_2_ separation, such as ZIFs,^14^ Cu-MOFs,^15^ Al-MOFs,^7^ and Zr-MOFs.^16^ Among them, MIL-101(Cr), a Cr-based MOF composed of chromium ions and terephthalic acid ligands, holds significant potential for gas adsorption applications. MIL-101(Cr) possesses unsaturated Lewis acid sites, an exceptionally high specific surface area, large pore size, significant pore volume, superior separation selectivity, ease of regeneration, and excellent thermal, chemical, and water stability.^17^

While numerous experimental studies investigated the adsorption of individual N_2_ and CH_4_ gases using various MIL-101 modifications such as MIL-101(Cr),^18,19^ CH_3_- MIL-101(Cr) and NO_2_-MIL-101(Cr),^20^ NH_2_-MIL101(Al),^21^ NH_2_-MIL101(Cr),^22^ and NH_2_-MIL101(Fe),^23^ few theoretical studies have been conducted to investigate this area. Zhang et al.^20^ utilized Density functional theory (DFT) to compare the electron cloud density and its effects on N_2_ adsorption for different functional group-modified MIL-101 structures, which ignored many key information on N_2_ adsorption mechanism, such as MIL-101 topological structure and molecular sieving effect. Sedighi et al.^24^ analyzed N_2_ adsorption sites using snapshots and RDF through molecular simulation, but they did not analyze from the RDF that the C=C bond of the linker of MIL-101 is also one of the adsorption sites and did not quantitatively compare the effects of different adsorption sites. Teo et al.^25^ analyzed the spatial distribution of CH_4_ in MIL-101 from snapshots, the analysis with only snapshots was not very comprehensive to understand CH_4_ adsorption in MIL-101. Although Liu et al.^26^ performed molecular simulation to explore the spatial distribution of CH_4_ in MIL-101, they did not explain the reason why CH_4_ has such a distribution. Additionally, they used a united-atom model with one site to represent CH_4_, which ignored the impact of molecular configuration (e.g., C–H bonds,^27^ molecular space orientations, octopole moment^28^) on the spatial distribution during mutation. And there was no quantitative analysis of which atomic structures played a major and secondary role in the adsorption of CH_4_.

Yoon et al.^29,30^ reported that the trend of preferential adsorption of CH_4_ versus N_2_ on MIL-101 reverses, when the preheating temperature of MIL-101(Cr) synthesis process was increased to 523 K. These studies did not provide an in-depth analysis of adsorption mechanism for CH_4_ and N_2_. For example, Yoon et al. did not describe the details about how to analyze the adsorption sites of CH_4_ in the entire text. Thus, it remains open to debate whether the conclusion is fully substantiated that the primary adsorption site for CH_4_ is in the proximity of metallic element Cr. Furthermore, to align with their special experimental findings, Yoon et al.^30^ computed new force field parameters specifically tailored to match the experimental data. By adjusting the force field parameters for N_2_, Yoon et al. examined that the primary adsorption site for N_2_ is in close proximity to metallic element Cr. However, it is important to note that this assertion may not hold true universally. Yoon et al. also used snapshots to observe the distance between the metal and N_2_ to identify the main adsorption sites. This analytical approach appears to be heavily biased or lacking in considering multiple perspectives. Additionally, the studies of Yoon et al. did not quantitatively compare the effects of different adsorption sites and ignored the influence of the special topological structure of MIL-101(Cr) on the adsorption of CH_4_ and N_2_.

Therefore, these theoretical studies failed to consider the influence of the molecular radius and MIL-101(Cr) topology structure of CH_4_ and N_2_ on the adsorption behavior and lacked a quantitative analysis of the significant and secondary roles played by specific atomic structures in the adsorption of CH_4_ and N_2_. As a result, a comprehensive understanding of the detailed adsorption behavior of CH_4_ and N_2_ on MIL-101 is still required.

Limited experimental research has been conducted on the utilization of MIL-101(Cr) for the adsorption and separation of CH_4_/N_2_ mixtures. Zhang et al. conducted experiments on the synthesis of MIL-101(Cr) and investigated its N_2_ and CH_4_ adsorption equilibrium and CH_4_/N_2_ separation capabilities.^31^ The experimental data demonstrated that the N_2_ and CH_4_ adsorption isotherms conformed to the Langmuir model. The selectivity for CH_4_/N_2_ separation ranged from 2.6 to 3.3, varying with changes in the temperature and pressure. Another study by Li et al. examined the impact of Mg^2+^ doping on the pore structure of MIL-101 and its adsorption selectivity for CH_4_/N_2_ gas mixtures.^32^ The findings indicated that Mg^2+^ doping enhanced the adsorption capacity of both CH_4_ and N_2_, as well as the selectivity for CH_4_/N_2_ separation. This effect was attributed to the inhibition of hydrogen bond formation resulting from the appropriate amount of Mg^2+^ doping, which positively influenced CH_4_ adsorption. Furthermore, Zhang et al. explored the modification of MIL-101 through the addition of −NO_2_ and −CH_3_ functional groups to alter the selectivity of CH_4_/N_2_ separation.^20^ Experimental results revealed that −NO_2_ exhibited a greater separation effect on CH_4_/N_2_ than −CH_3_, with a selectivity of 2.8 at 298 K and 1 bar. Additionally, the diffusion of molecules in porous materials plays an important role in many chemical processes, such as shape selective catalysis, molecular sieving, and selective adsorption. With better understanding of the complexities of gas diffusion in porous materials, it is necessary for the design, development, and optimization of catalysis and adsorption.^33^ In adsorption processes, diffusion governs the rate of adsorption–desorption cycles in adsorption-based processes and directly impacts the selectivity of products in gas separations.^34^ So, there is a lack of further explanation of mixture diffusion and adsorption at the microscopic level.

In this study, the primary objective is to employ grand canonical Monte Carlo (GCMC) and molecular dynamics (MD) simulation methods to gain a comprehensive understanding of the influence of structural variations in the MIL-101 framework on the adsorption and diffusion of CH_4_ and N_2_ gases within its pores. The research aims to address three fundamental questions, as illustrated in Figure S1: (I) What are the adsorption and diffusion behaviors of single-component CH_4_ and N_2_ in MIL-101? (II) How do the adsorption and diffusion behaviors differ in the presence of two-component CH_4_ and N_2_ in MIL-101? (III) Are the adsorption behaviors of the single- and two-component systems interconnected?

Accordingly, this article is divided into two parts: the analysis of the single-component system and the analysis of the two-component system. Before the analysis in Parts 1 and 2, for single-component gases, it is necessary to verify the reliability of the simulation and validate the force filed through the adsorption isotherm and isosteric heat. In the case of the two-component system, the reliability of the simulation is assessed by analyzing the selectivity of CH_4_/N_2_, which enables a more comprehensive investigation of the adsorption and diffusion behaviors. The analysis primarily utilizes three methods: density distribution profile, self-diffusion coefficients, and RDF. By elucidating the mass transport characteristics of CH_4_ in MIL-101 with varying N_2_ concentrations at the atomic level, this study provides valuable theoretical insights for the separation of CH_4_/N_2_ mixtures using MIL-101(Cr).

## 2 Methods

### 2.1 Grand Canonical Monte Carlo Simulations

**Figure 1:**
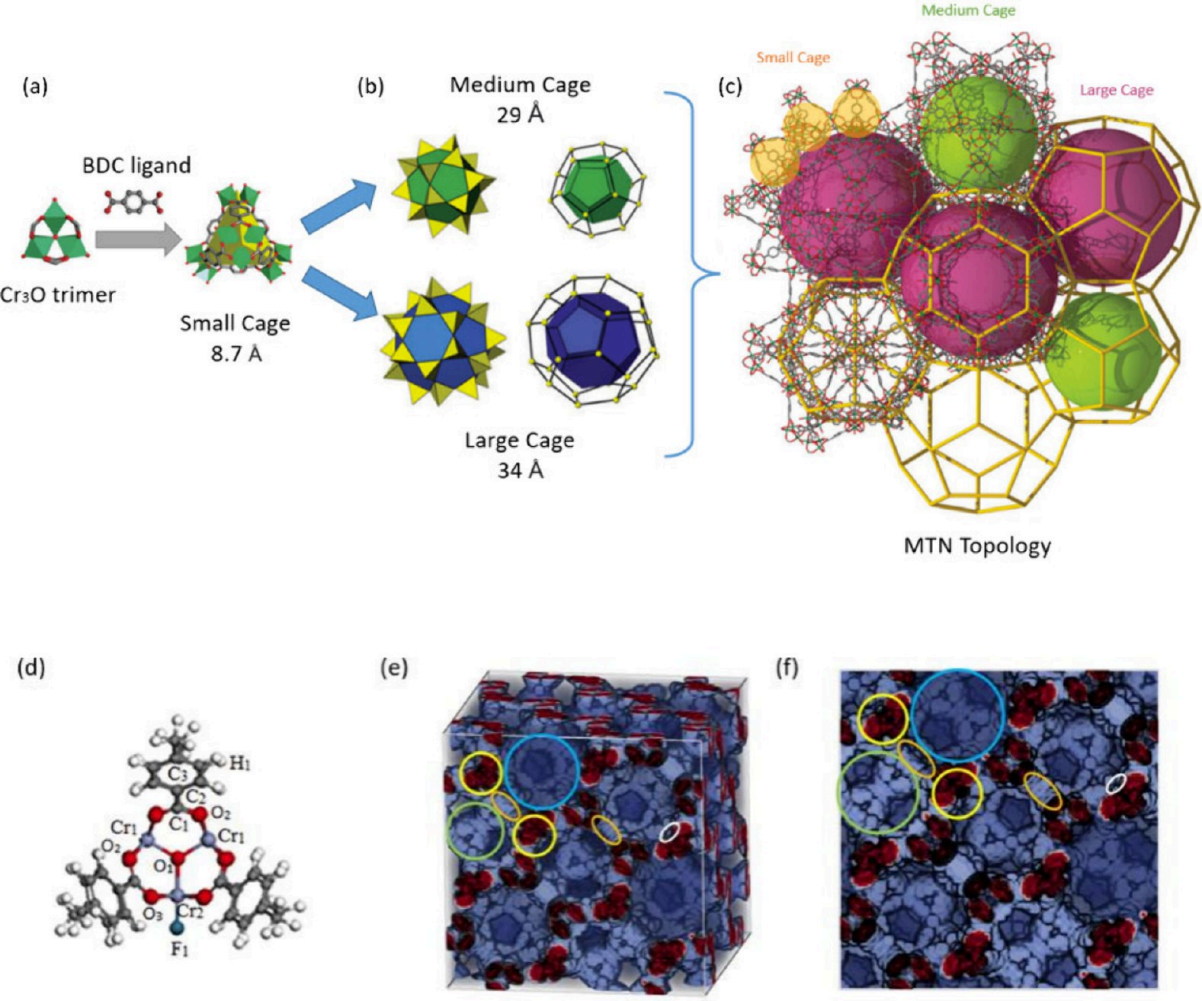
(a) Tetrahedral cage of MIL-101; (b) medium and large cage of MIL-101;^35^ (c) topology of MIL-101;^36^ (d) atom labels of MIL-101;^24^ (e) 3D view of simulation cell; and (f) 2D view of simulation cell (large cage: blue; medium cage: green; small cage: yellow; pentagon window: orange; and triangle window: white).

To investigate the adsorption of CH_4_ and N_2_, and their mixture in MIL-101(Cr), the RASPA2 platform,^37^ a molecular simulation software program, was employed. The grand canonical Monte Carlo (GCMC) approach was utilized, where the adsorbate chemical potential, volume, and temperature were kept constant during the simulations. The structure of MIL-101(Cr) (https://github.com/iRASPA/RASPA2/blob/master/structures/mofs/cif/MIL-101.cif) depicted in Figure 1 was considered as rigid in these simulations. Within the simulation cell, CH_4_ and N_2_ molecules were allowed to exhibit various forms of motion including swapping, rotation, and translation. Lennard-Jones (L-J) potential functions were employed to model the van der Waals interactions between the CH_4_ and N_2_ molecules and the atoms of the MIL-101(Cr) framework.^37^
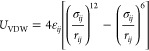
1When *σ*_*ij*_ is the finite distance at which the interparticle potential is zero, *ε*_*ij*_ is the depth of potential well, *r*_*ij*_ is the distance between the particles *i* and *j*, and the Lorentz–Berthelot rules can be used to determine the “strength” and “size” parameters that describe the potential as follows
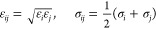
2

Coulombic interactions between charged particles were modeled via the Ewald approach.^37^
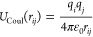
3where ε_0_ is the dielectric permittivity of a vacuum and *q*_*i*_ and *q*_*j*_ are the respective partial charges of atoms *i* and *j*.

The GCMC method considers the chemical potential, volume, and temperature as independent variables within the μVT ensemble. In this ensemble, the chemical potential is held constant, while the temperature and system volume are fixed. It is worth noting that the chemical potential is typically determined based on fugacity rather than pressure. Those GCMC calculations were conducted at a temperature of 298 K and a fixed pressure ranging from 0.1 to 1 bar. The L-J 12-6 potential calculation employed a cutoff radius of 12 Å. The van der Waals interactions were computed using an atom-based summation approach, while the electrostatic interactions between the listed atoms were evaluated using the Ewald summation method. During the GCMC simulations, MIL-101(Cr) frameworks with 1 × 1 × 1 unit cells (88.869 × 88.869 × 88.869 Å^3^) were utilized, and periodic boundary conditions were applied in all three dimensions. The equilibration phase involved the first 5000 moves, and ensemble averages were computed using the final 25,000 steps. All simulations included random insertion/deletion, translation, and rotation moves of molecules with equal relative probabilities, which are properly normalized within the RASPA2 code. So they only need to be specified relative to each other.^37^ Detailed information regarding the L-J potential parameters and atomic charges for each labeled atom can be found in Table 1. The CH_4_ molecule was represented using a rigid five-site model,^38^ where partial charges and L-J potential parameters were assigned to both the C and H atoms to replicate their electrostatic characteristics and van der Waals interactions. The partial charges on the C and H atoms were derived from calculation of the octopole moment. Specifically, the partial charge on each hydrogen atom *q*_H_ is 0.165*e*, and the partial charge on the carbon atom *q*_C_ is −0.66*e*, where *e* represents the magnitude of the electronic charge. The N_2_ molecule was described by a rigid 3-site with partial charges located on the N and N_com_ atoms of N_2_ to reproduce its electrostatic potential and a Lennard-Jones site located on the N atom.^24^ The L-J parameters of the atoms in MIL-101 are all from the DREIDING^39^ and UFF^40^ force fields, and the partial charges of the atoms are all from the work of Dubbeldam et al.^37^ To aid in comprehending the structural information (e.g., 3D topological view, 3D density distribution profile, volume visualization mode, atom visualization mode), we utilized iRASPA,^41^ a structural visualization tool that facilitated the visualization of the simulated results.

**Table 1:** L-J Potential Parameters and Charges of CH_4_ and N_2_ and MIL-101(Cr)^24,38−40,42^

			L-J parameter		
Molecule	Unique identifier	Atom	ε/*k*_B_ (K)	σ (Å)	Charge/e
CH_4_	CAS RN: 74-82-8	C_CH_4_	55.055	3.40	–0.66
		H_CH_4_	7.901	2.65	0.165
N_2_	CAS RN: 7727-37-9	N_N_2_	36.00	3.31	–0.48
		N_com^a^	–	–	0.96
MIL-101(Cr)	cif file name: MIL-101(Cr)	O1	48.16	3.03	–0.85
		O2	48.16	3.03	–0.57
		O3	48.16	3.03	–0.44
		C1^24^	52.87	3.43	0.50
		C2^24^	52.87	3.43	–0.07
		C3^24^	52.87	3.43	–0.06
		H1^24^	22.14	2.57	0.11
		F1^42^	36.48	3.09	–0.55
		Cr1	7.55	2.69	1.62
		Cr2	7.55	2.69	1.35

a N_com represents massless virtual site, placed at the molecular center of mass.

### 2.2 Molecular Dynamics Simulations

To investigate the self-diffusion properties of guest molecules within MIL-101, we employed MD simulations using the LAMMPS software.^43^ The simulations were conducted in both the canonical ensemble (NVT) and microcanonical ensemble (NVE) to examine the behavior of gas molecules within the different cavities of MIL-101(Cr). For those MD simulations, MIL-101 frameworks with 1 × 1 × 1 unit cells (88.869 × 88.869 × 88.869 Å^3^) were utilized, and periodic boundary conditions were applied in all three dimensions. The L-J potential was employed to model the intermolecular interactions with a cutoff radius of 12 Å. This is because cutoff radius for 12 Å is based on empirical experience of LAMMPS with the Leonard Jones interaction in the pair_style command^44^ (e.g., pair_style lj/cut/coul/long 12). The parameters for the interaction between atoms of different species, which are not explicitly provided, were determined using the customary Lorentz-Bertelot combining rules (e.g., pair_modify mix arithmetic). The Particle-Particle Particle-Mesh (PPPM) summation approach was used to calculate the long-range electrostatic interactions with a precision of 1 × 10^–4^ (e.g., kspace_style pppm 0.0001). The tail correction^45^ was used for computing the long-range interactions of certain pairs during the simulations (e.g., pair_modify tail yes). Newton’s laws of motion were integrated into the equation of motion using the Velocity-Verlet technique. The simulation time step was set to 0.2 fs. Each MD simulation consisted of a total duration of 11 ns, with 10 ns allocated for equilibration and 1 ns for production to ensure a stable state and achieve complete statistical averaging.^46,47^ A Nose-Hoover thermostat with a damping coefficient of 100 time steps at 300 K was employed to control the temperature. The MD simulations considered the flexibility of the MIL-101 framework but treated CH_4_ and N_2_ as rigid bodies. As shown in Table S3, the force field parameters for MIL-101(Cr) flexible framework were calculated from LAMMPS Interface.^48^ Such approach has been widely utilized for MOFs with flexible frameworks.^49^

## 3 Results and Discussion

### 3.1 Adsorption of Pure CH_4_ and N_2_

#### 3.1.1 Simulation Validation of Pure Gases

**Figure 2:**
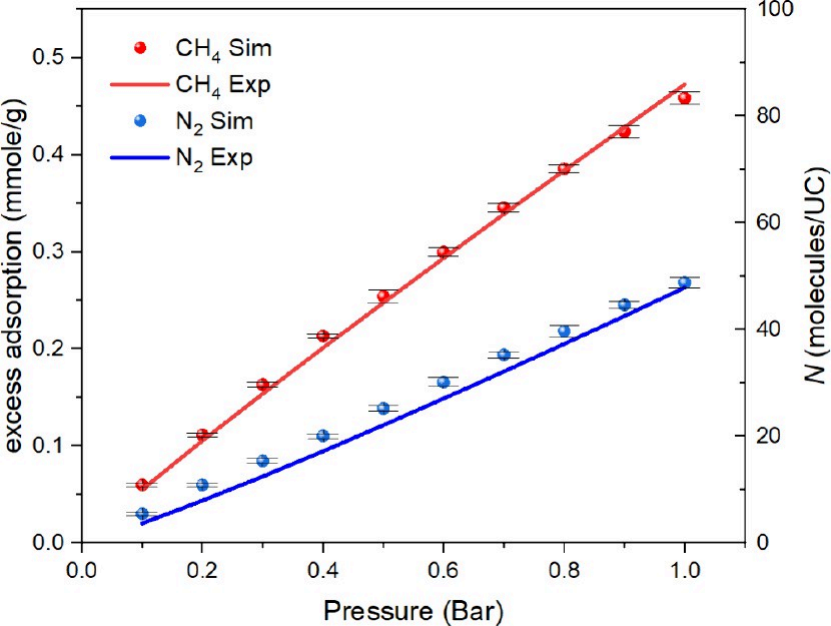
CH_4_^19^ and N_2_^50^ adsorption isotherms comparison of GCMC simulations and experimental measurements in MIL-101(Cr) at 298 K.

The simulated adsorption isotherms of CH_4_ and N_2_ on MIL-101(Cr) at 298 K were compared with experimental data to validate the simulation results.^19,50^Figure 2 and Table S1 show that the simulated adsorption isotherms were slightly higher than the values obtained from the experimental data. This can be attributed to the absence of crystal defects or impurities in the MIL-101(Cr) model used in the simulations, which may have a minor influence on the adsorption capacity. Figure 2 also reveals that the adsorption capacities of both CH_4_ and N_2_ increase with increasing pressure. However, the rate of increase for CH_4_ is noticeably higher than that for N_2_. Additionally, the adsorption capacity of CH_4_ is higher than that of N_2_ under the investigated conditions. It can be also obtained from Figure 2 and Table S1 that under the pressure range of 0.1–1 bar, one unit cell of MIL-101(Cr) is capable of adsorbing between 11 and 86 CH_4_ molecules and between 6 and 50 N_2_ molecules, respectively. These findings suggest that MIL-101 exhibits better adsorption performance for CH_4_ compared to N_2_, particularly at low pressures.

The isosteric heat of adsorption (*Q*_st_) is a crucial parameter that reflects the strength of the interaction between an adsorbent and an adsorbate, particularly at low pressures or low loading conditions. In this study, the *Q*_st_ values for CH_4_ and N_2_ adsorption on each MOF at different operating temperatures were estimated by using the GCMC method. The calculation of *Q*_st_ was performed using eq 4 as described in the literature.^42^
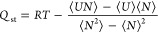
4where *U* is the change in internal energy of intermolecular interactions in the adsorbed phase during the adsorption process, *N* is the molecular weight of the adsorption, *R* is the gas constant, and ⟨ ⟩ is the average value. The complete derivation process for eq 4 can be found in the Supporting Information.

**Figure 3:**
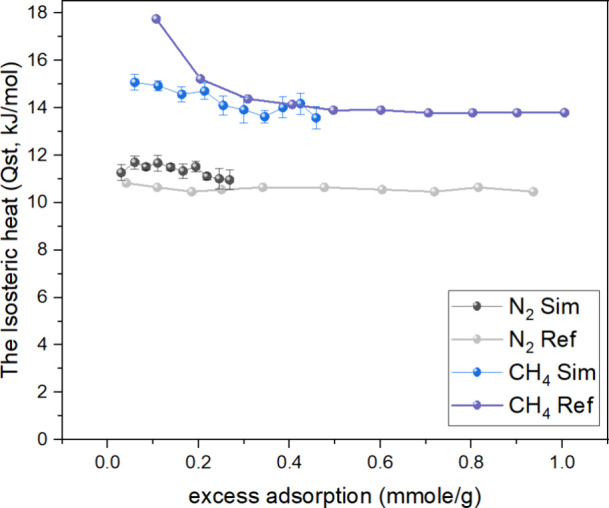
Isosteric heat of adsorption (*Q*_st_) for N_2_^51^ and CH_4_^52^ in MIL-101.

The fluctuation of the isosteric heat of adsorption for CH_4_ and N_2_ on MIL-101 at 298 K is presented in Figure 3 and Table S2. The *Q*_st_ values for CH_4_ are consistent with previous findings reported by Chowdhury et al.,^52^ who reported values of approximately 14–18 kJ/mol. However, it is important to note that at low loading, our simulation results indicate a value of around 15.08 kJ/mol, which deviates slightly from their findings (17.75 kJ/mol). As the loading increases, the enthalpy of adsorption for CH_4_ shows a slight decrease, ranging from approximately 15.08 kJ/mo at 0.06 mmol/g to around 13.57 kJ/mol at 0.46 mmol/g. This observation can be attributed to the dominant role of van der Waals forces and electrostatic interactions in the low-load region, while in the high-load region, as the adsorption sites become saturated, the *Q*_st_ decreases.

For N_2_, the simulation outcomes closely align with the experimental results reported by Zhang et al.^51^ The enthalpy of adsorption for N_2_ exhibits only slight variations with loading. Notably, the *Q*_st_ values for CH_4_ are significantly higher than those for N_2_, indicating stronger interactions between CH_4_ molecules and the MIL-101 framework. The accuracy and reliability of our simulation results for both the adsorption isotherms and *Q*_st_ values have been confirmed, thus validating the usage of the GCMC simulation method in the grand canonical ensemble for further research.

#### 3.1.2 Radial Distribution Functions of Pure Gases

The RDF is mainly used for point-to-point quantitative research on which atoms in the adsorbent play a major role in gas adsorption, so as to know which adsorption sites in the adsorbent and which atoms the adsorption sites are composed of.^53^ This function is to count the number of two-atom species at a given distribution distance, which can be specified as^49^
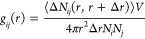
5where *V* is the system volume, *N*_*i*_ and *N*_*j*_ are the numbers of atoms *i* and *j*, respectively, and Δ*r* is the distance between atoms *i* and *j*. *N*_*ij*_(*r, r + r*) is the number of atom *j* surrounding *i* within a shell from *r* to *r* + Δ*r*.

**Figure 4:**
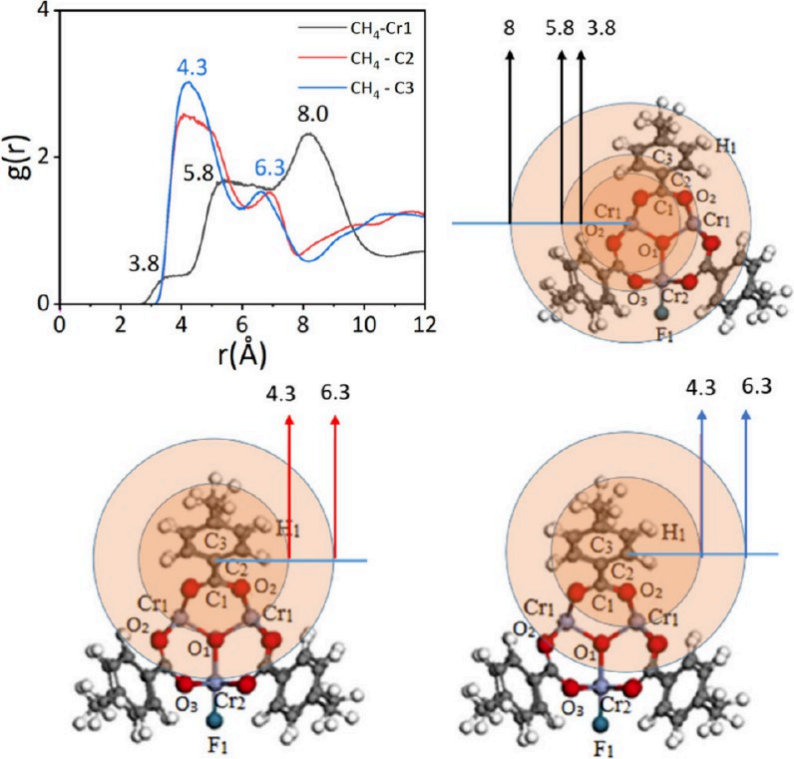
RDF of CH_4_ around the Cr1 atom and C3 atom.

In Figure 4, the RDF curve for Cr1 exhibits three distinct peaks at approximately 3.8, 5.8, and 8 Å. The highest peak, located at 8 Å, indicates that CH_4_ molecules tend to accumulate at this distance from the Cr1 atom. This accumulation is primarily influenced by the presence of C2 and C3 (C=C) atoms at the 8 Å distance, which facilitates the interaction between CH_4_ and the framework. On the other hand, the peak observed at 5.8 Å shows a lower density of CH_4_ molecules compared to the peak at 8 Å. This can be attributed to the increased steric hindrance effect^54^ near 5.8 Å, which hinders further interaction between CH_4_ and the C2/C3 atoms. The presence of nearby atoms and their spatial arrangement influence the accessibility and binding of CH_4_ molecules at this distance. Furthermore, the peak observed at 3.8 Å is the result of the synergistic effect of multiple atoms, including O2, O1, and Cr1, on the adsorption of CH_4_ at this distance. The combined influence of these atoms contributes to the favorable adsorption of CH_4_ molecules at a 3.8 Å distance. These findings highlight the important role of the C=C bond in MIL-101 and its effect on the adsorption of CH_4_. The study conducted by Qin et al.^55^ supports these observations, further emphasizing the significance of the C=C double bond in facilitating the adsorption of CH_4_ in MIL-101.

In Figure 4, there are peaks observed around 4.3 and 6.3 Å in the C2 and C3 curves. The highest peak at approximately 4.3 Å indicates that the majority of CH_4_ molecules are adsorbed near the C=C bond in the MIL-101 framework. This suggests that the C=C bond plays a crucial role in the adsorption of CH_4_ molecules as it provides favorable interaction sites. Additionally, a smaller peak is observed around 6.3 Å, which can be attributed to the interaction between the Cr and O atoms in the MIL-101 framework. This interaction contributes to a smaller amount of CH_4_ being adsorbed at this distance. Furthermore, it is noteworthy that the peak at 4.3 Å in the C3 curve is higher than that in the C2 curve. This difference can be explained by considering the structural differences around the C2 and C3 atoms. CH_4_ molecules surrounding the C3 atom experience less steric hindrance compared to those surrounding the C2 atom, allowing for a higher density of adsorbed CH_4_ molecules at the 4.3 Å distance. These findings provide insights into the specific adsorption sites and the role of the C=C bond and Cr and O atoms in the adsorption of CH_4_ molecules in MIL-101.

The RDF diagrams of CH_4_ for different types of atoms, as shown in Figure S2a–d, provide strong evidence of the significant influence of the C=C double bond on the adsorption of CH_4_. The presence of Cr and O atoms also contributes to the adsorption process. The variation in peak height observed in the RDF diagrams can be attributed to two main factors. First, the distance from the adsorption site plays a role, as different locations of atoms can result in variations in peak heights. Second, steric hindrance effects can weaken the van der Waals force, leading to variations in the strength of adsorption at different distances. These findings reinforce the importance of the C=C double bond in CH_4_ adsorption, while also highlighting the involvement of Cr and O atoms in the overall adsorption mechanism.

There are also related studies of CH_4_ adsorption on MOFs (e.g., ZIF-8, ZIF-76, ZIF-69, MIL-101(Cr)) that have reached similar conclusions, supporting our research results. For example, Pérez-Pellitero et al.^56^ reported that based on their molecular simulation, preferential adsorption sites for CH_4_ in ZIFs (e.g., ZIF-8, ZIF-76, ZIF-69) are located in specific regions close to the organic imidazolate linker, and not on the metal atoms. This contrasts with the behavior of several MOFs in which the preferential adsorption site is not on the organic linker but on the metal center. Additionally, Wu et al.^57^ reported that using neutron powder diffraction and difference Fourier analysis, they experimentally determined that the primary methane adsorption sites are associated with the organic linkers in ZIF-8. Meanwhile, Zhang et al.^58^ reported that at a low loading, CH_4_ primarily occupies the adsorption site proximal to the C=C bond of 2-methylimidazolate. Therefore, their study showed that the organic linker in ZIF-8 is the most preferential adsorption site rather than the metal cluster. Furthermore, Qin et al.^55^ reported that through DFT calculation for the analysis of the adsorption site of MIL-101(Cr), these gases (e.g., CH_4_, C_2_H_6_, C_3_H_8_) are not adsorbed on the open metal site, which attracts negative charged atoms while the H of alkanes are positively charged. Their DFT calculations showed that the C–H···π distances exhibit a discernible trend of shortening as the gas molecule increases in size. Taking the shortest C–H···π distances as an example, they range from 3.70 to 3.14 Å for MIL-101-Cr, 3.70 to 3.16 Å for MIL-101-Fe, and 3.52 to 2.97 Å for MIL-101-Fe-NH_2_, respectively. Simultaneously, C–H···O distances either decrease or increase. These findings underscore the critical role of C–H···π interactions.

The RDF results of Cr, O, C, and F for N_2_ in Figure S3 show similarities to their respective RDF spectra for CH_4_. This suggests that the adsorption behavior of N_2_ and CH_4_ is influenced by similar atomic sites within the MIL-101 framework.

#### 3.1.3 Density Distribution Profile of Pure Gases

The primary application of 2D and 3D density distribution contours is to study the spatial distribution of gases in an adsorbent, with a specific emphasis on the point-to-volume topological relationship. These plots offer a more intuitive visualization of how gases are distributed throughout various channels and pores within the adsorbent.

**Figure 5:**
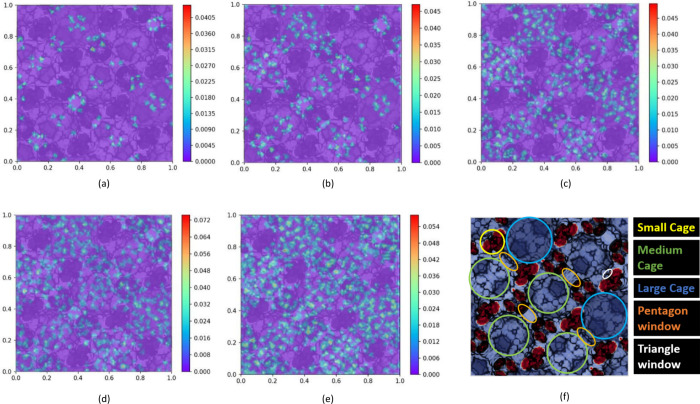
2D density distribution for CH_4_ in MIL-101 with different numbers of CH_4_ molecules: (a) number = 100, (b) number = 200, (c) number = 400, (d) number = 600, (e) number = 800, and (f) 2D view of simulation cell.

**Figure 6:**
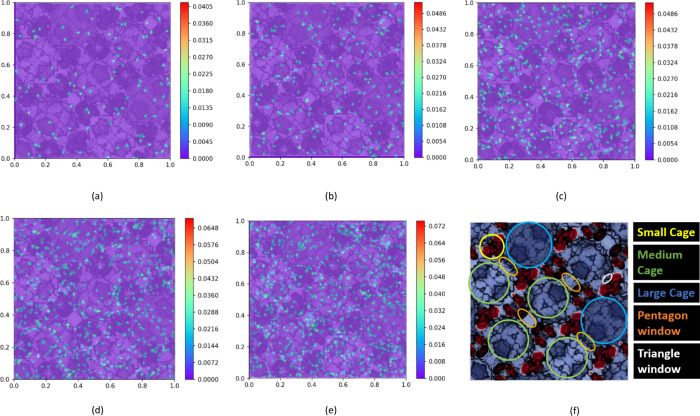
2D density distribution for N_2_ in MIL-101 with different numbers of N_2_ molecules: (a) number = 100, (b) number = 200, (c) number = 400, (d) number = 600, (e) number = 800, and (f) 2D view of simulation cell.

Figure 5 and 6 depict the 2D density distribution profile of N_2_ and CH_4_ in MIL-101 with different molecule numbers projected onto the *xy* plane at 300 K, respectively. It can be seen from Figure 5 that when the number of CH_4_ molecules is 100, CH_4_ is mainly distributed in pentagonal windows and large and medium cages. With the increase of the number of CH_4_ molecules, the large and medium cages are gradually filled by CH_4_ molecules, but as the number of CH_4_ molecules reaches 800, few CH_4_ molecules begin to appear in the small cages of the tetrahedron, and the large and medium cavities were almost covered with CH_4_ molecules. The place with the highest density of CH_4_ is on the edges of the pentagonal window connecting the large and medium cages. This shows that CH_4_ has a high probability of adsorption on the edge of the pentagonal window.

However, the N_2_ density profile is obviously different from that of CH_4_. It can be clearly seen from Figure 4 that when the number of N_2_ molecules is 100, most of the N_2_ molecules are relatively uniformly distributed on the edges of the pentagonal window and the small cages of the tetrahedron, and a small amount of N_2_ molecules appear inside the tetrahedron. As the number of N_2_ molecules increases, N_2_ molecules are gradually distributed in large, medium, and small pores, but they do not appear in the same distribution pattern as CH_4_. Most CH_4_ gathers in medium and large pores, and very few appear in small pores. Instead, the highest N_2_ density occurs in small tetrahedral cavities. It illustrates that N_2_ has a high probability of adsorption in small cages.

**Figure 7:**
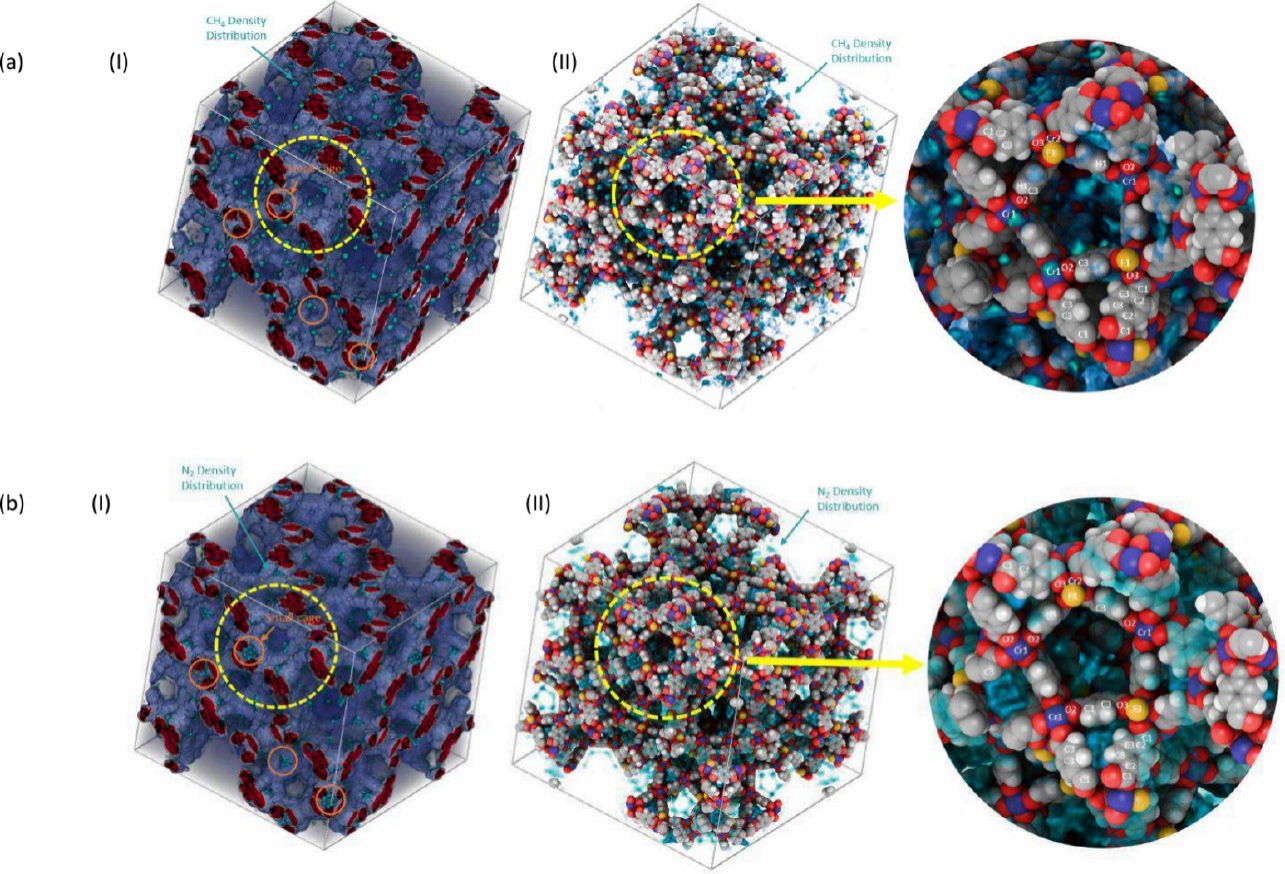
(a) 3D density distribution for CH_4_, (b) 3D density distribution for N_2_, (I) volume visualization mode, and (II) atoms visualization mode.

In order to better understand their adsorption mechanisms, the 3D density distribution profile can be seen to which atoms the molecules of CH_4_ and N_2_ are adsorbing. The distribution of N_2_ molecules and CH_4_ molecules in MIL-101 can be seen more clearly from Figure 7a.I and b.I. The area circled by orange is the small cavity of the tetrahedron. It can be clearly seen that there are very few CH_4_ molecules in the tetrahedral cavity, but there are many N_2_ molecules in that. So it can be concluded that N_2_ is more likely to be adsorbed in small cavities than CH_4_.

It can be seen from Figure 7a.II that CH_4_ molecules are mainly distributed on the edge of the pentagonal window rather than the triangle window. As illustrated in Figure 7a.II, the highest concentration of CH_4_ is found at the C=C bond of the benzene ring, close to the C3 and C2 atoms. Few CH_4_ molecules appear near the trimer of the Cr and O atoms. The results observed by the 3d density distribution profile are basically consistent with the results analyzed by the RDF spectrum. So it shows that C=C double bond and unsaturated metal (Cr) are the main adsorption sites of CH_4_ molecules.

However, the 3d density distribution of N_2_ is obviously different from that of CH_4_. It can be seen from Figure 7b.II that N_2_ molecules are mainly distributed near the tetrahedron (including the edge and body), and few are distributed on the edge of the pentagonal window. It is also observed that the main interactive atoms in MIL-101 are also near the C=C double bond on the benzene ring and the trimer of unsaturated metal (Cr).

The structure and availability of the pores in MIL-101 are currently being examined. Morphologies and channel diameters within the material have been calculated utilizing the HOLE program,^59^ as presented in Table 2. Figure S4 reveals a triangular pore window with a diameter of 4.8 Å, slightly larger than the kinetic diameter of CH_4_ (3.8 Å). Due to the random movement of CH_4_ molecules, the probability of them passing through such a small window is extremely low, making the triangular pore window inaccessible to CH_4_. Conversely, N_2_ with a diameter of 3.0 Å, 20% smaller than that of CH_4_, has a higher probability of passing through the triangular pore window. The hexagonal and pentagonal pore windows, with diameters of 13.0 and 10.0 Å, respectively, are much larger than the critical diameter of CH_4_, allowing both CH_4_ and N_2_ to easily access the medium and large cages within MIL-101.

**Table 2:** Pore Windows and Pore Diameter of MIL-101 Calculated Using HOLE Program^59^

Type	Diameter (Å)
Large cage	32.0
Hexagonal pore window	13.0
Medium cage	28.0
Pentagonal pore window	10.0
Small cage	6.6
Triangle pore window	4.8

In summary, the density distribution profile indicates that N_2_ can be evenly distributed throughout MIL-101, while CH_4_ faces limitations due to the size difference between the molecules and the pore structure. The smaller size of N_2_ enables it to pass through smaller pore windows, resulting in a more uniform distribution. The pore structure of MIL-101 and the size of gas molecules are key factors determining their distribution patterns within the material.

#### 3.1.4 Self-Diffusion Coefficients and Diffusion Activation Energy of Pure Gases

The self-diffusion coefficient (*D*_s_) serves as a quantitative tool for analyzing adsorption kinetics. Its primary purpose is to compare the diffusion rates of different gases within an adsorbent, with a particular focus on the randomized Brownian motions of the gas itself influenced by the adsorbent.^60^ This differs from the analysis conducted by RDF and density distribution functions, which primarily focuses on examining the adsorption sites within the adsorbent.

The *D*_s_ of N_2_ and CH_4_ in MIL-101 at 298 K were investigated to understand their diffusion properties. The *D*_s_ values were determined by calculating the mean-squared displacement (MSD) of the molecules. The average MSD of molecule *j*, as described in eq 6, is directly related to the diffusion coefficients according to the Einstein equation. By analyzing the MSD, we can obtain valuable insights into the diffusion behavior of N_2_ and CH_4_ within MIL-101 at different loadings and concentrations.^49^
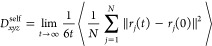
6where *N* is the number of molecules *j* in the simulation system, *r*(*t*) represents the position vector of molecule *j* at time *t*, and ⟨···⟩ denotes an ensemble average.

To calculate the self-diffusion coefficients of CH_4_ and N_2_ in MIL-101, a series of simulations were performed. The MIL-101 structure was equilibrated at 300 K through a 1 ns simulation in the NVT ensemble, followed by a 10 ns simulation in the NVE ensemble, to obtain the relaxed crystal structure. This procedure was conducted once for each set of framework parameters. Afterward, the desired number of CH_4_ and N_2_ molecules were loaded into the relaxed structure, and the system was further simulated for 10 ns in the NVT ensemble to reach equilibrium. Subsequently, a 1 ns production run was performed in the NVT ensemble.

**Figure 8:**
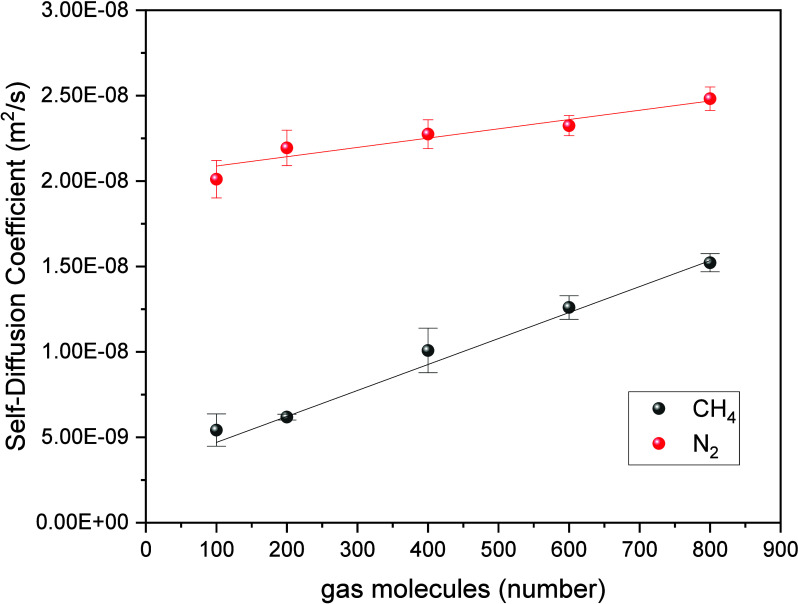
Self-diffusion coefficients of CH_4_ and N_2_ molecules in MIL-101(Cr).

**Figure 9:**
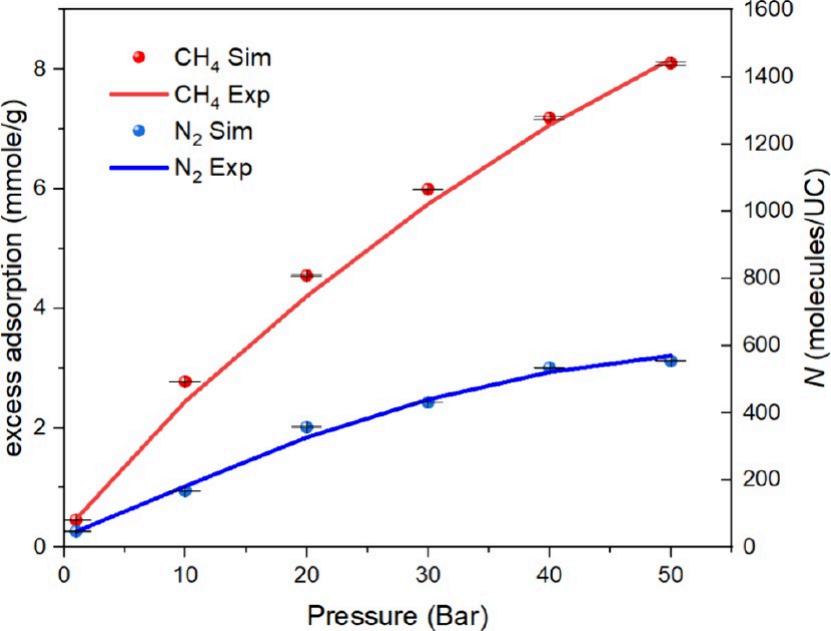
CH_4_^52^ and N_2_^61^ adsorption isotherms comparison of GCMC simulations and experimental measurements in MIL-101(Cr) at 298 K.

In Figure 8, we present a comparison of the self-diffusion coefficients of different numbers (e.g., 100, 200, 400, 600, 800) of CH_4_ and N_2_ molecules in MIL-101(Cr). The corresponding isotherm pressures of these numbers under GCMC simulations can be found in Figure 9 and Table S9. It can also be obtained that under the pressure range of 1–50 bar, one unit cell of MIL-101(Cr) is capable of adsorbing between 89 and 1524 CH_4_ molecules and between 50 and 582 N_2_ molecules, respectively. The calculated self-diffusion coefficients fell within the range from 10^–9^ to 10^–8^ m^2^/s, consistent with relevant studies by Sun et al.^62^ on the experimental measurement of CH_4_ gas diffusion coefficient (1.95 to 3.21 × 10^–9^ m^2^/s) in porous materials and Forse et al.^34^ on the experimental study of the effect of pore size on the gas diffusion (1.5 to 6 × 10^–9^ m^2^/s) in MOFs. Sedighi et al.^63^ reported a study on the diffusion behavior of N_2_ using modified MIL-101. Their study showed that the self-diffusion coefficient of N_2_ is concentrated in the range of 2 to 6 × 10^–8^. Additionally, Zhang et al.^58^ used molecular simulation to examine adsorption and diffusion of CH_4_ in ZIF-8. Their study showed that the value of the self-diffusion coefficient of CH_4_ obtained from Zhang et al. is in the range of 10^–10^ to 10^–9^. Figure 8 and Table S4 illustrate an increasing trend in the self-diffusion coefficients of both CH_4_ and N_2_, which agreed well with previous work of Sedighi et al.^63^ and Zhang et al.^58^ This can be attributed to the nearly constant number of adsorption sites in MIL-101, indicating that the atoms in MIL-101 exerting effective forces on CH_4_ and N_2_ remain unchanged. Consequently, when the number of CH_4_ and N_2_ molecules is low, the average force on each molecule is relatively higher, resulting in stronger binding and lower self-diffusion coefficients. Moreover, the self-diffusion coefficient of N_2_ is higher than that of CH_4_, indicating that N_2_ exhibits easier diffusion, confirming that CH_4_ is more readily adsorbed by MIL-101.

The impact of temperature on CH_4_ and N_2_ diffusion is depicted in Figure S5a, revealing a linear increase in self-diffusivity as temperature rises. This trend is consistent with similar observations in other microporous materials.^64,65^ The enhanced movement of gas molecules at higher temperatures leads to an increase in their diffusion coefficients. Figure S5b presents the Arrhenius plots of CH_4_ and N_2_ diffusion parameters; there are many studies^66−68^ using this method to get the activation energy of diffusion and commonly use to characterize gas diffusion in porous materials. According to the classical Arrhenius equation, the self-diffusion coefficient (*D*_s_) can be calculated as *D*_s_ = *D*_0_*e*^–*E*_a_/*RT*^, where *E*_a_ is the activation energy of diffusion (kJ/mol), *D*_0_ is the pre-exponential factor, *R* is the universal gas constant (8.314 J/mol K), and *T* is the temperature (K). By plotting the inverse temperature (1/*T*) against the natural logarithm of the self-diffusion coefficient (ln(*D*_s_)), the slope of the curve provides information about the activation energy. Figure S5b demonstrates a good linear relationship (*R*^2^ = 0.9701 and 0.9625) between ln(*D*_s_) and 1/*T*, consistent with the equation . The slope of the curve allows computation of the diffusion activation energies. In this case, the diffusion activation energies for CH_4_ and N_2_ in MIL-101 were determined to be 8.07 and 4.40 kJ/mol, respectively. A lower activation energy suggests a faster diffusion process, with a smaller migration barrier to overcome. The higher activation energy for CH_4_ compared to N_2_ indicates that N_2_ diffuses more easily than CH_4_ in MIL-101.

### 3.2 Adsorption of CH_4_/N_2_ Mixture

#### 3.2.1 Simulation Validation of CH_4_/N_2_ Binary Mixture

**Figure 10:**
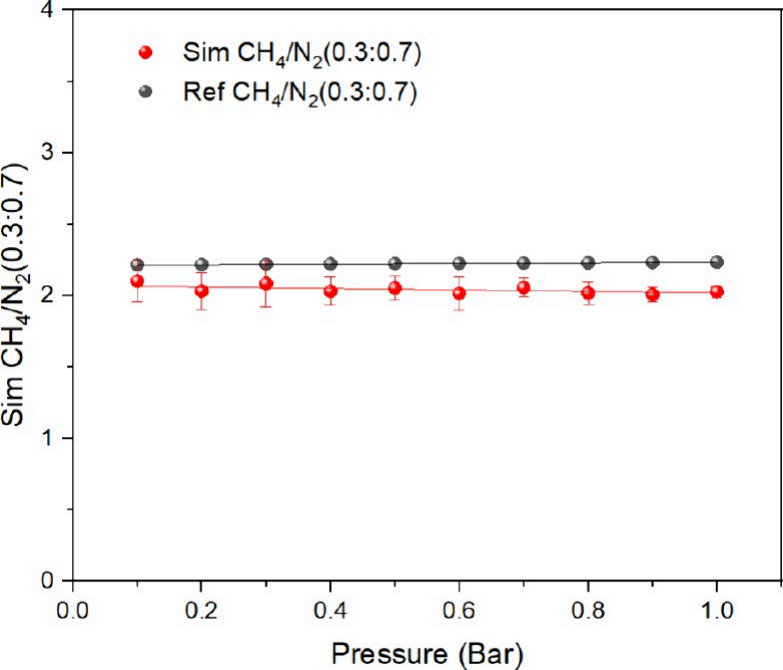
Separation selectivity values of CH_4_/N_2_ (0.3:0.7) mixture for adsorption on MIL-101(Cr) at 298 K.

The GCMC method is used to determine the selectivity values of the CH_4_/N_2_ (0.3/0.7)^32^ gas mixture in MIL-101 at 298 K and under various pressures. We set this ratio to the concentration ratio of the initial gas phase. The predicted selectivity values for the adsorption of CH_4_ over N_2_ for MIL-101 are shown in Figure S8. The separation selectivity equation^26^ is used to compute the data as shown below:
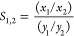
7where *x* and *y*, for components 1 and 2, denote the equilibrium adsorbed and gas phase mole fractions, respectively. A follow-up study on the adsorption of the adsorbent MIL-101 for the mixed two-component gas (CH_4_ and N_2_) behavior and adsorption mechanism, as well as adsorption sites, is needed to validate the simulated value with the experimental value of selectivity in order to increase the simulation’s credibility.^32^ It can be seen from Figure 10 and Table S8 that the simulated results are very close to the experimental values, and the simulated values are slightly higher than the experimental values. The main reason is that simulations have traditionally emphasized “ideal” crystals, but actual samples inevitably exhibit imperfections, ranging from minor defects to the interpenetration and localized collapse of the structure.

#### 3.2.2 Density Distribution Profiles and Snapshot of CH_4_/N_2_ Binary Mixtures

**Figure 11:**
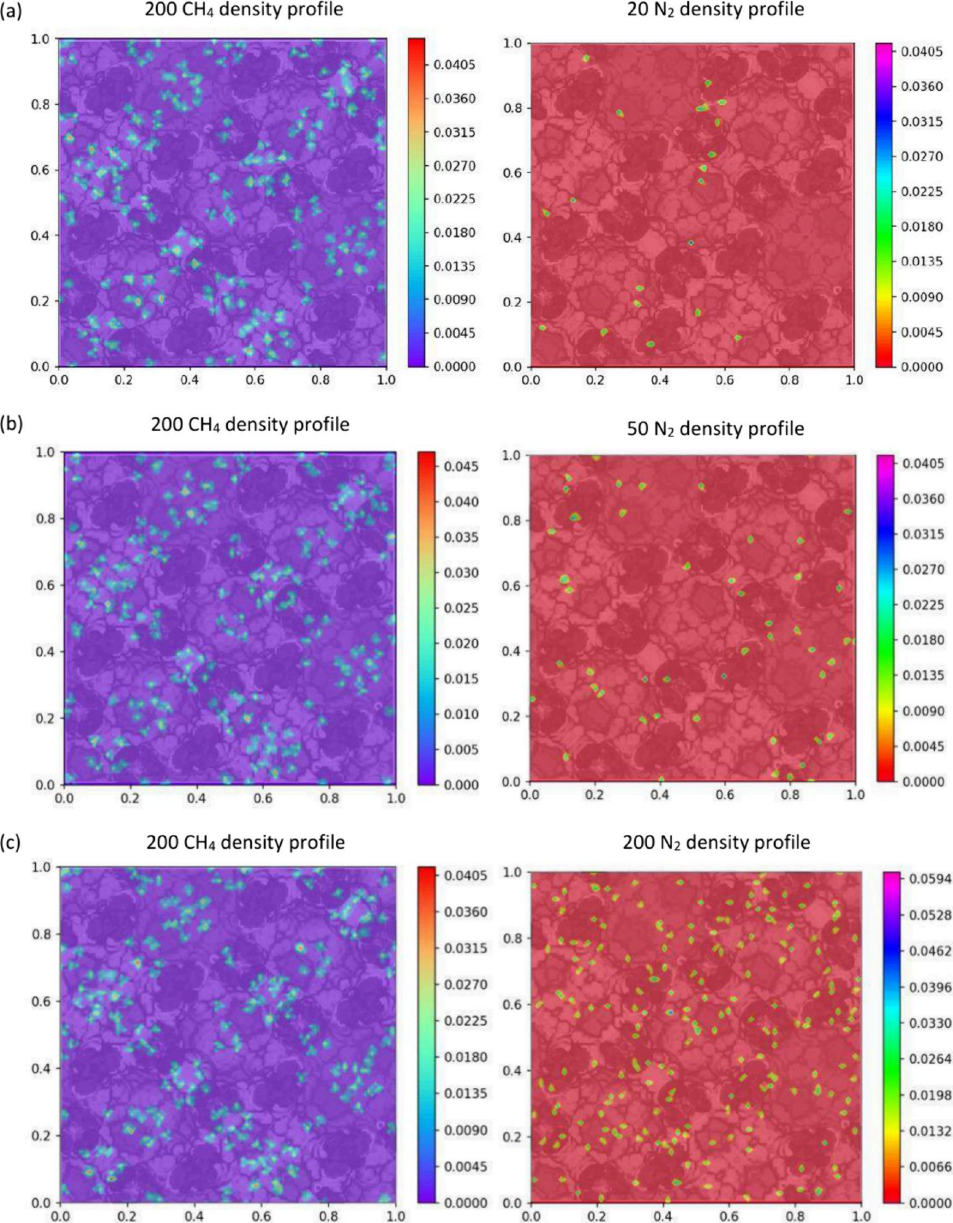

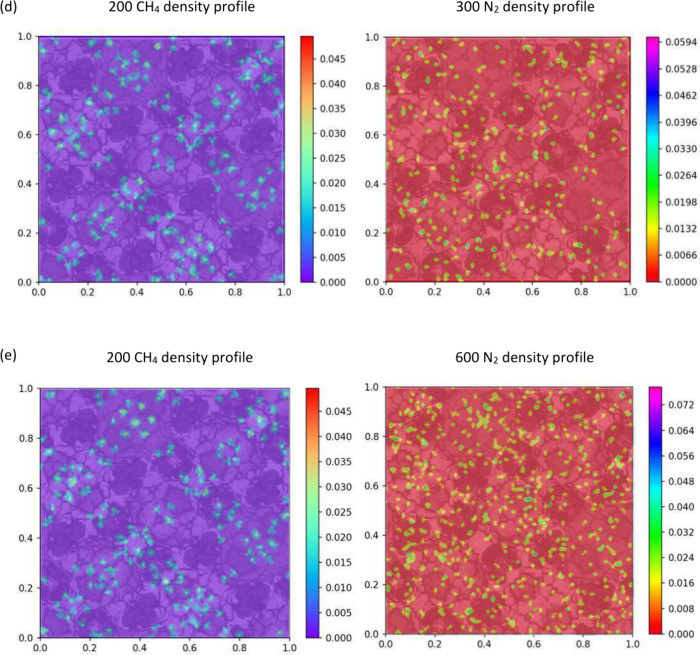
2D density distribution of CH_4_ and N_2_ for 200 CH_4_ molecules in MIL-101 with different numbers of N_2_ molecules: (a) number = 20, (b) number = 50, (c) number = 200, (d) number = 300, and (e) number = 600.

Figure 11 illustrates the 2D density distribution profiles of CH_4_ with varying numbers of N_2_ molecules within MIL-101. In Figure 11a, it can be observed that the 200 CH_4_ molecules are primarily distributed in the large- and medium-sized cages, with some molecules also located near the pentagonal window. Notably, certain regions within the adsorbent exhibit the clustering of CH_4_ molecules. On the other hand, the 20 N_2_ molecules tend to be concentrated near the small tetrahedral cavities and triangular windows, as depicted in Figure 11a.

Figure 11b–e demonstrate that, as the number of N_2_ molecules increases, the overall distribution of CH_4_ molecules remains largely unaffected. The previous distribution pattern is maintained, and there is no apparent similarity between the distribution patterns of N_2_ and CH_4_. With the addition of more N_2_ molecules, N_2_ tends to exhibit a more even distribution throughout the adsorbent without significant aggregation within individual cages. The highest density of N_2_ molecules appeared near the tetrahedron. Conversely, CH_4_ molecules maintain their distribution pattern without a pronounced influence from the increasing number of N_2_ molecules.

Furthermore, Figure 11 reveals that the highest density values of CH_4_ increase as the number of N_2_ molecules increases. This can be attributed to the fact that when the overall number of CH_4_ molecules remains constant, the introduction of more N_2_ molecules leads to partial compression of CH_4_ molecules, resulting in an increase in the highest density of CH_4_ molecules. Simultaneously, the highest density of N_2_ also exhibits an increment in response to the growing number of N_2_ molecules, corresponding to the overall increase in the total N_2_ molecule count.

**Figure 12:**
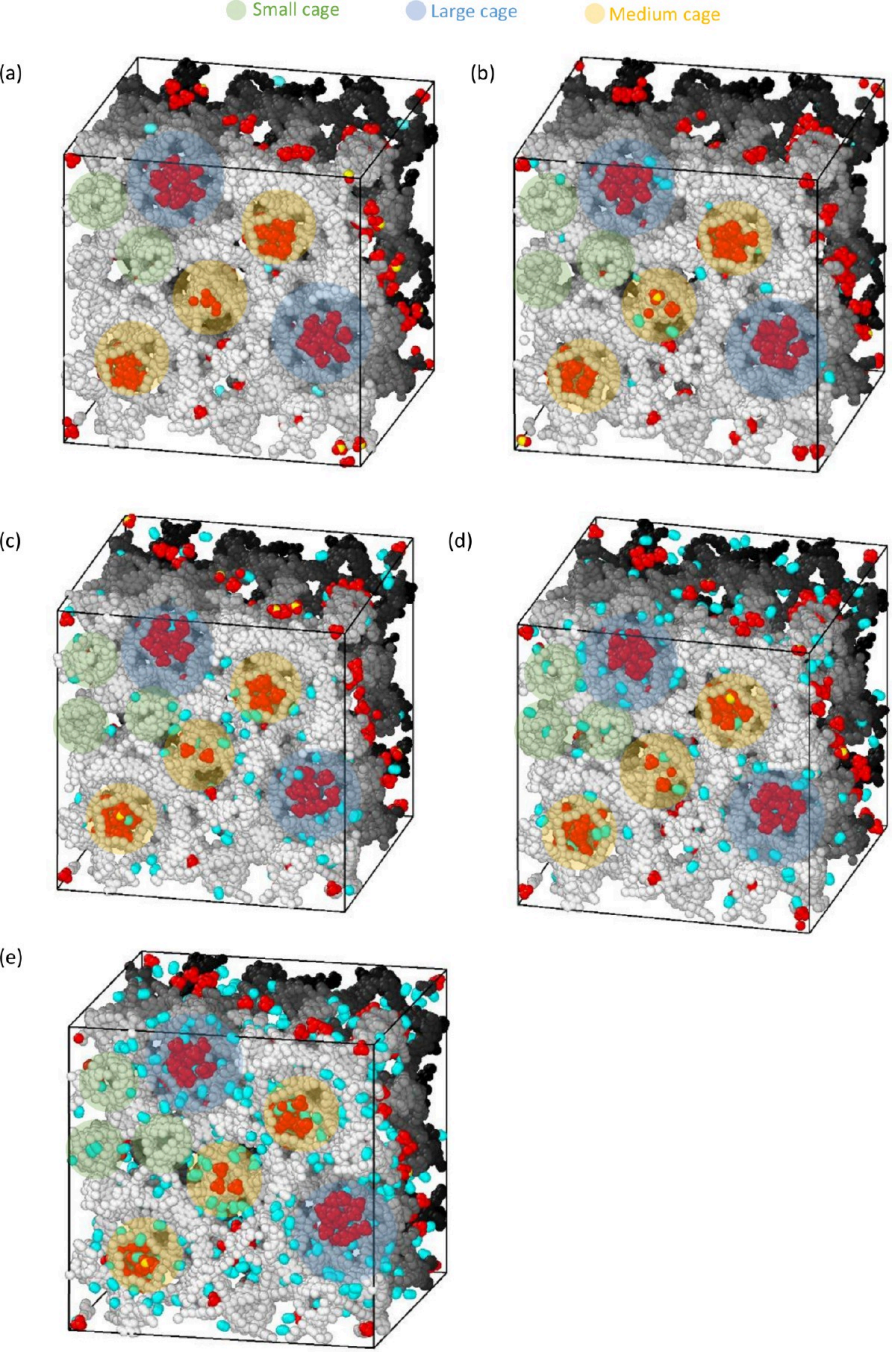
Snapshot for 200 CH_4_ molecules in MIL-101 with different numbers of N_2_ molecules: (a) number = 20, (b) number = 50, (c) number = 200, (d) number = 300, and (e) number = 600 (CH_4_: red and yellow; N_2_: blue).

Figure 12 depicts a snapshot of the distribution of 200 CH_4_ molecules and different numbers of N_2_ molecules in the adsorbent MIL-101. Through the display of 3D snapshots, the distribution of gas molecules can be seen more intuitively. As the increase of N_2_ molecules does not affect the distribution of CH_4_ molecules, it can be clearly seen that CH_4_ molecules are agglomerated in large and medium-sized cages, and it can be clearly seen that N_2_ molecules tend to distribute on the edges of large and medium-sized cages and windows and also appear in the small cavity.

#### 3.2.4 Self-Diffusion Coefficients of CH_4_/N_2_ Binary Mixture

**Figure 13:**
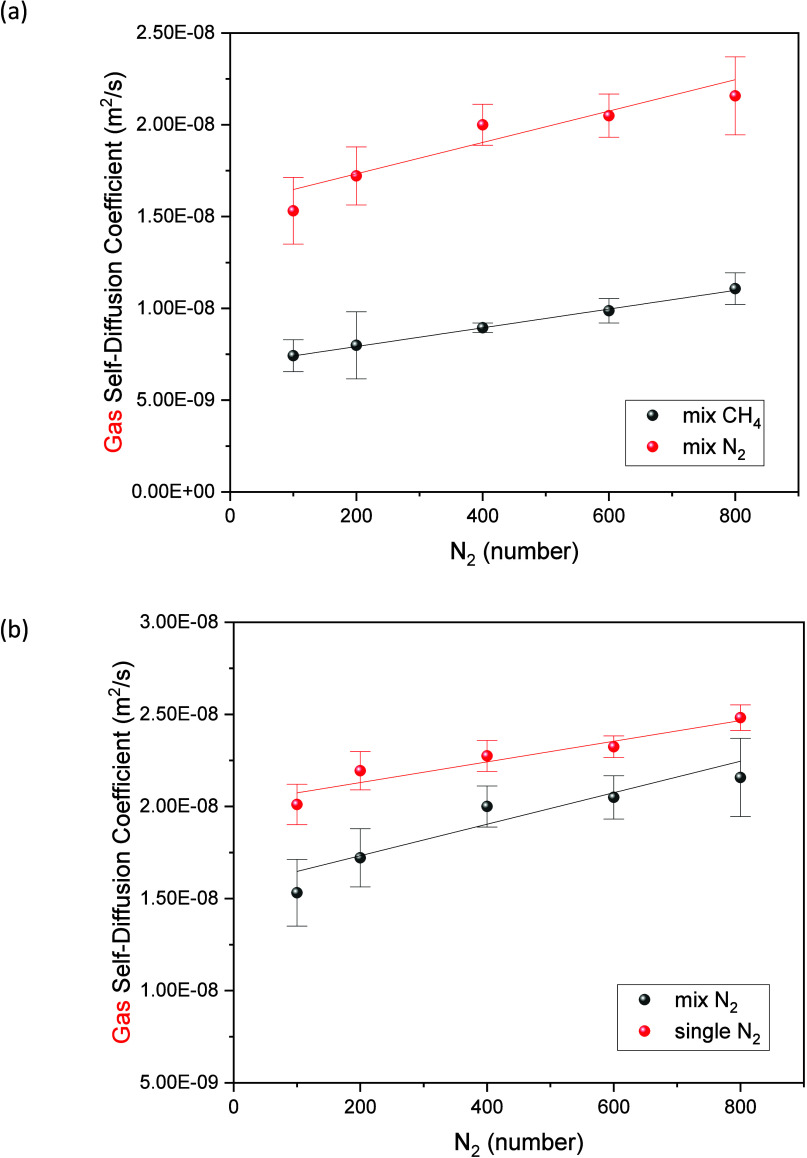
(a) Self-diffusion coefficients of CH_4_ and N_2_ when loading 200 CH_4_ molecules in MIL-101 at different loadings of N_2_ molecules at 298 K. (b) Self-diffusion coefficients of N_2_ when compared loading 0 CH_4_ molecules with 200 CH_4_ molecules at different loadings of N_2_ molecules at 298 K.

Figure 13a and Table S6 present the impact of increasing the number of N_2_ molecules on the self-diffusion coefficient of each component in the binary mixture system of CH_4_ and N_2_. As depicted in Figure 13a, the self-diffusion coefficients of N_2_ and CH_4_ exhibit a corresponding increase as the number of N_2_ molecules increases in the binary gas mixture system. This behavior can be attributed to the fact that the total number of adsorption sites of the adsorbent is constant, and the increase in the number of gas molecules in the system will occupy more adsorption sites, the resulting reduction in the force exerted by the adsorbent on the newly added gas molecules leads to an increase in the diffusion coefficient. It is also worth noting that the self-diffusion coefficient of N_2_ is higher than that of CH_4_. This discrepancy arises from the higher selectivity of MIL-101 for CH_4_ compared to that of N_2_. The adsorbent exhibits a preference for adsorbing CH_4_ molecules, leading to a greater occupation of adsorption sites by CH_4_. Consequently, the van der Waals and Coulomb forces exerted by the adsorbent on N_2_ are diminished, resulting in a relatively higher self-diffusion coefficient for N_2_.

Additionally, we conducted a comparison between the self-diffusion coefficient of N_2_ in the absence of CH_4_ molecules and in the presence of 200 CH_4_ molecules, as shown in Figure 13b and Table S7. It is evident that with an increase in the number of N_2_ molecules, both the self-diffusion coefficients of N_2_ with and without CH_4_ molecules increase. This can be attributed to a limited number of available adsorption sites for the newly introduced gas molecules.

The Figure 13b also shows that the self-diffusion coefficient of N_2_ in the presence of 200 CH_4_ gas molecules is significantly lower than that of pure N_2_. When the concentration of N_2_ is low, CH_4_ exerts a greater hindrance on N_2_ diffusion. However, as the concentration of N_2_ increases, the difference between the self-diffusion coefficient of N_2_ in the mixed gas and that in pure gas diminishes. This reduction in difference is primarily due to the decreasing proportion of CH_4_ in the mixture, leading to a reduced hindrance effect on the N_2_ diffusion. Such hindrance effect has been observed in many studies of CO_2_/CH_4_,^69^ CO_2_/N_2_,^70^ CO_2_/Ar,^71^ and CH_4_/N_2_^71^ in porous materials. Therefore, we can conclude that in MIL-101(Cr), the presence of CH_4_ assists in reducing the self-diffusion of N_2_.

## 4 Conclusions

This study employs GCMC and MD simulations to investigate the adsorption and diffusion behavior of CH_4_, N_2_, and their mixture in the MIL-101(Cr) adsorbent. Several properties were computed and examined, including adsorption isotherms at varying temperatures, self-diffusion coefficients, isosteric heat of adsorption, density distribution profiles, radial distribution functions, and CH_4_/N_2_ selectivity values within the MIL-101 framework.

The results were consistent with the anticipated outcomes.

(1) The calculations of self-diffusion coefficient and isosteric heat of adsorption indicate that N_2_ exhibits a higher self-diffusion coefficient compared to CH_4_, suggesting that N_2_ diffuses more easily. Moreover, both gases display a linear increase in the self-diffusion coefficient with temperature. The diffusion activation energy calculations confirm that N_2_ has a lower diffusion activation energy than CH_4_, indicating its greater ease of diffusion. These results shed light on the transport properties of CH_4_ and N_2_ within the MIL-101 framework. (2) The CH_4_/N_2_ selectivity calculations align well with experimental data, underscoring the adsorbent’s preference for CH_4_ molecules. Additionally, the analysis of 2D density distribution profiles for binary mixtures reveals that the overall distribution of CH_4_ molecules remains largely unaffected by an increase in the number of N_2_ molecules.

In addition to the anticipated outcomes, the following is a summary of the novel and original findings.

The RDF analysis reveals that the C=C bond in MIL-101 is crucial for the adsorption of CH_4_ and N_2_, while the Cr and O atoms contribute to a lesser extent. Examination of the 2D density distribution profiles shows that CH_4_ molecules preferentially occupy pentagonal windows and large and medium cages, with the highest density observed at the edges of the pentagonal window connecting the large and medium cages. Similarly, N_2_ molecules exhibit a relatively uniform distribution along the edges of pentagonal and triangular windows as well as in the small cages of the tetrahedron.

The 3D density distribution analysis highlights that the main interactive atoms in MIL-101 are located near the C=C double bond on the benzene ring and the trimer of the unsaturated metal (Cr). These insights provide valuable information about the dominant adsorption sites and molecular arrangements within the adsorbent. The isosteric heat of adsorption is determined to be approximately 14 kJ/mol for CH_4_ and 11 kJ/mol for N_2_, respectively, indicating differing adsorption strengths for the two gases.

In the CH_4_/N_2_ selectivity analysis, the presence of CH_4_ affects the N_2_ diffusion trajectory, resulting in a self-diffusion coefficient of N_2_ in the mixed gas that is smaller than that of single-component N_2_. Moreover, the introduction of more N_2_ molecules causes some CH_4_ molecules to be compressed together, leading to a change in the highest density value of CH_4_.

These findings lay the groundwork for potential modifications aimed at enhancing the adsorption and selectivity properties of MIL-101 for the selective capture of coal-mine methane. The molecular simulation approaches employed in this study provide a precise understanding of the adsorption and desorption mechanisms within the MIL-101 framework.

## Supporting Information Available

Workflow diagram, adsorption isotherm comparisons, isosteric heat of adsorption, RDF comparisons, MIL-101 pore diameter through the channel, self-diffusion coefficients, effects of temperature, force field parameters, uncertainty analysis, and calculation of isoteric heat of adsorption (DOCX)
