# Supplementary material for: Adsorption and Diffusion of CH4, N2, and Their Mixture in MIL-101(Cr): A Molecular Simulation Study
Source: J Chem Eng Data. 2024 Aug 22;69(12):4466–82. doi: 10.1021/acs.jced.4c00233 (PMC11647892; doi:10.1021/acs.jced.4c00233)
Supplement: Supplementary file 1 — je4c00233_si_001.docx [file je4c00233_si_001.docx]

**SUPPORTING INFORMATION**

**Adsorption and Diffusion of CH_4_, N_2_ and their Mixture in MIL-101(Cr): A Molecular Simulation Study**


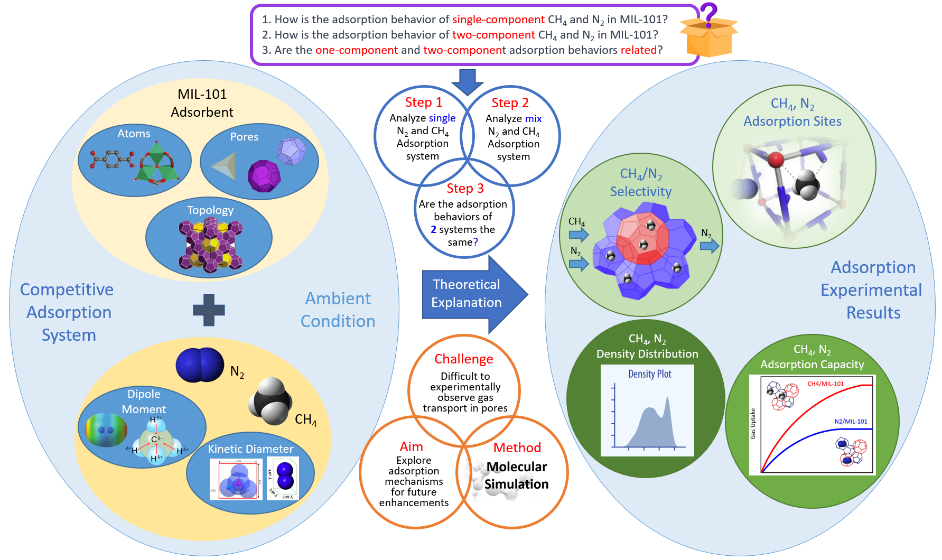


Figure S1. Workflow diagram of research question, steps, methods and purpose.

Table S1. CH_4_  ^1^ and N_2_ ^2^ adsorption isotherms***^α^*** comparison of GCMC simulations and experimental measurements in MIL-101(Cr) at 298 K.

| Pressure (Bar) | CH_4_ - MIL101(Cr) | | | N_2_- MIL101(Cr) | | |
| --- | --- | --- | --- | --- | --- | --- |
|  | **CH_4_ Exp** | **CH_4_ Sim** | | **N_2_ Exp** | **N_2_ Sim** | |
|  | excess  adsorption  (mmole/g) | excess adsorption (mmole/g) | Number  (molecules  /UC) | excess  adsorption  (mmole/g) | excess adsorption (mmole/g) | Number  (molecules  /UC) |
| 0.10 | 0.0553 | **0.0598** (0.0016) | 11 | 0.0174 | **0.03001** (0.00149) | 6 |
| 0.20 | 0.1055 | **0.1113** (0.0024) | 21 | 0.0521 | **0.05982** (0.00153) | 11 |
| 0.30 | 0.1539 | **0.1632** (0.0022) | 30 | 0.0782 | **0.08445** (0.00243) | 16 |
| 0.40 | 0.2013 | **0.2134** (0.0020) | 40 | 0.0955 | **0.11033** (0.00229) | 21 |
| 0.50 | 0.2478 | **0.2542** (0.0067) | 47 | 0.1216 | **0.13869** (0.00262) | 26 |
| 0.60 | 0.2937 | **0.2998** (0.0045) | 56 | 0.1390 | **0.16582** (0.00472) | 31 |
| 0.70 | 0.3391 | **0.3456** (0.0047) | 64 | 0.1650 | **0.19386** (0.00341) | 36 |
| 0.80 | 0.3840 | **0.3856** (0.0041) | 72 | 0.2084 | **0.21841** (0.00625) | 41 |
| 0.90 | 0.4286 | **0.4241** (0.0063) | 79 | 0.2345 | **0.24543** (0.00387) | 46 |
| 1.00 | 0.4728 | **0.4587** (0.0065) | 86 | 0.2605 | **0.26832** (0.00529) | 50 |

***^α^***The values in parentheses represent the uncertainty values.

Table S2 Isosteric heat of adsorption (*Q_st_*) ***^α^*** for N_2_ ^3^ and CH_4_ ^4^ in MIL-101.

| N_2_- MIL101(Cr) | | | | CH_4_ - MIL101(Cr) | | | |
| --- | --- | --- | --- | --- | --- | --- | --- |
| N_2_ Sim | | [**N_2_ Ref**](https://pubs.rsc.org/en/content/articlelanding/2013/ce/c3ce41737a) | | **CH_4_ Sim** | | [**CH_4_ Ref**](https://doi.org/10.1016/j.micromeso.2011.11.022) | |
| Excess adsorption (mmole/g) | **Isosteric heat (Qst, kJ/mol)** | **Excess adsorption (mmole/g)** | **Isosteric heat (Qst, kJ/mol)** | **Excess adsorption (mmole/g)** | **Isosteric heat (Qst, kJ/mol)** | **Excess adsorption (mmole/g)** | **Isosteric heat (Qst, kJ/mol)** |
| 0.03001 (0.00149) | **11.26848** (0.33582) | 0.0415 | 10.8333 | **0.0598** (0.0016) | **15.08092** (0.33206) | 0.106971 | 17.75685 |
| 0.05982 (0.00153) | **11.7047** (0.2746) | 0.1092 | 10.6482 | **0.1113** (0.0024) | **14.93304** (0.21847) | 0.204179 | 15.22356 |
| 0.08445 (0.00243) | **11.51434** (0.12345) | 0.1852 | 10.4630 | **0.1632** (0.0022) | **14.57425** (0.32284) | 0.308591 | 14.38130 |
| 0.11033 (0.00229) | **11.67213** (0.34212) | 0.2509 | 10.5556 | **0.2134** (0.0020) | **14.70788** (0.34549) | 0.406119 | 14.14270 |
| 0.13869 (0.00262) | **11.49639** (0.13887) | 0.3412 | 10.6482 | **0.2542** (0.0067) | **14.10748** (0.41136) | 0.496678 | 13.90389 |
| 0.16582 (0.00472) | **11.33141** (0.30163) | 0.4768 | 10.6482 | **0.2998** (0.0045) | **13.91887** (0.54141) | 0.601208 | 13.90704 |
| 0.19386 (0.00341) | **11.5225** (0.21409) | 0.6041 | 10.5556 | **0.3456** (0.0047) | **13.62955** (0.26549) | 0.705721 | 13.78942 |
| 0.21841 (0.00625) | **11.11584** (0.14359) | 0.7191 | 10.4630 | **0.3856** (0.0041) | **14.01956** (0.44456) | 0.803282 | 13.79237 |
| 0.24543 (0.00387) | **11.0143** (0.42899) | 0.8156 | 10.6482 | **0.4241** (0.0063) | **14.17878** (0.44627) | 0.900844 | 13.79532 |
| 0.26832 (0.00529) | **10.96239** (0.41087) | 0.9367 | 10.4630 | **0.4587** (0.0065) | **13.57704** (0.47744) | 1.005374 | 13.79847 |

***^α^***The values in parentheses represent the uncertainty values.


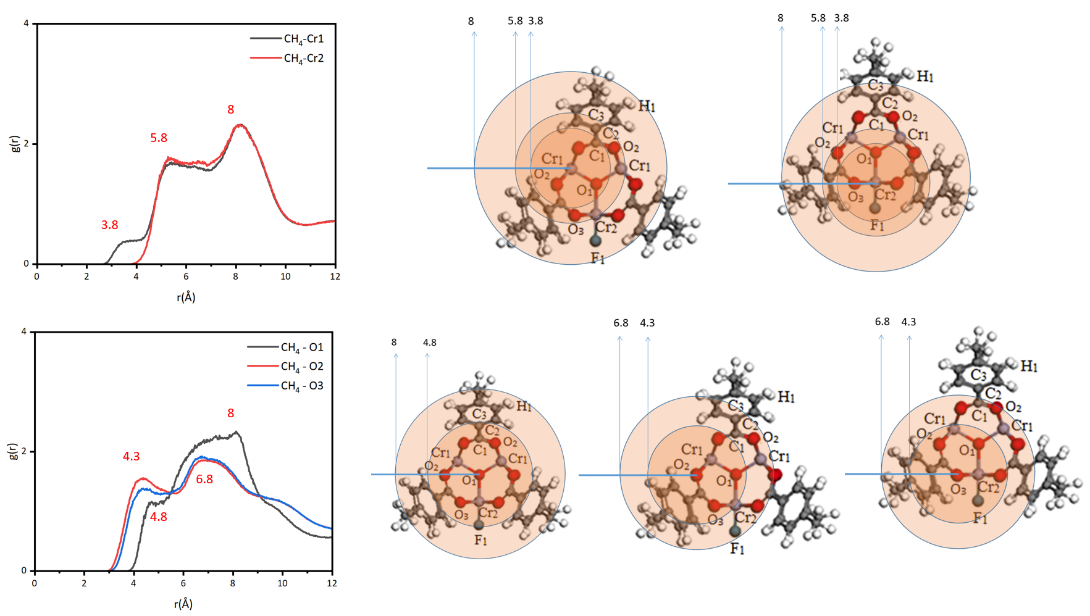


(a)

(b)

(c)


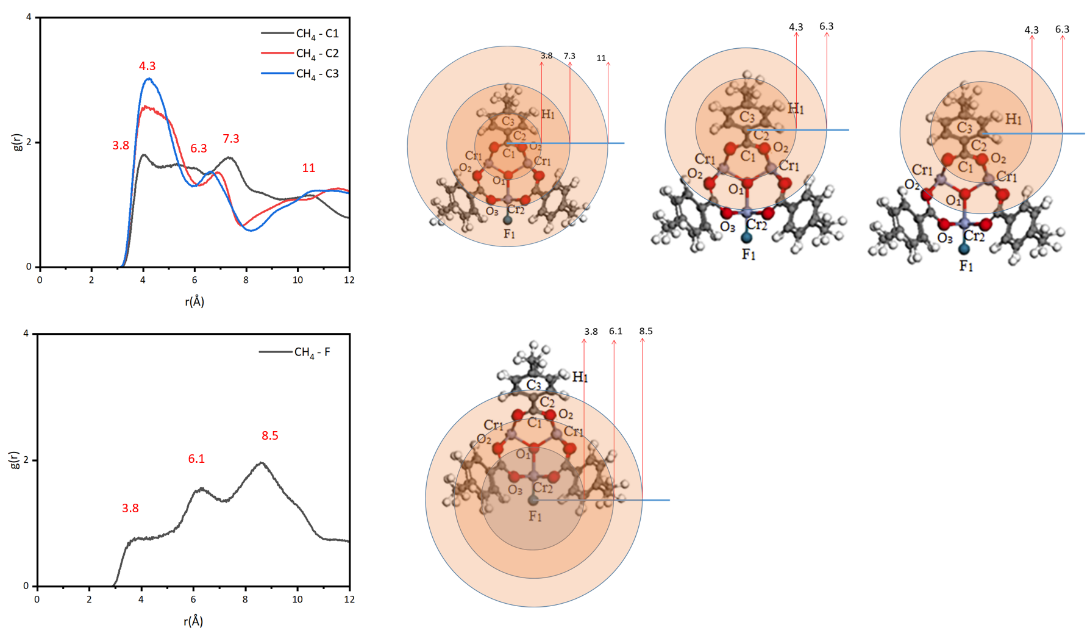


Figure S2. RDF of CH_4_ around (a) Cr atoms; (b) O atoms; (c) C atoms; (d) F atoms in MIL-101.

(d)


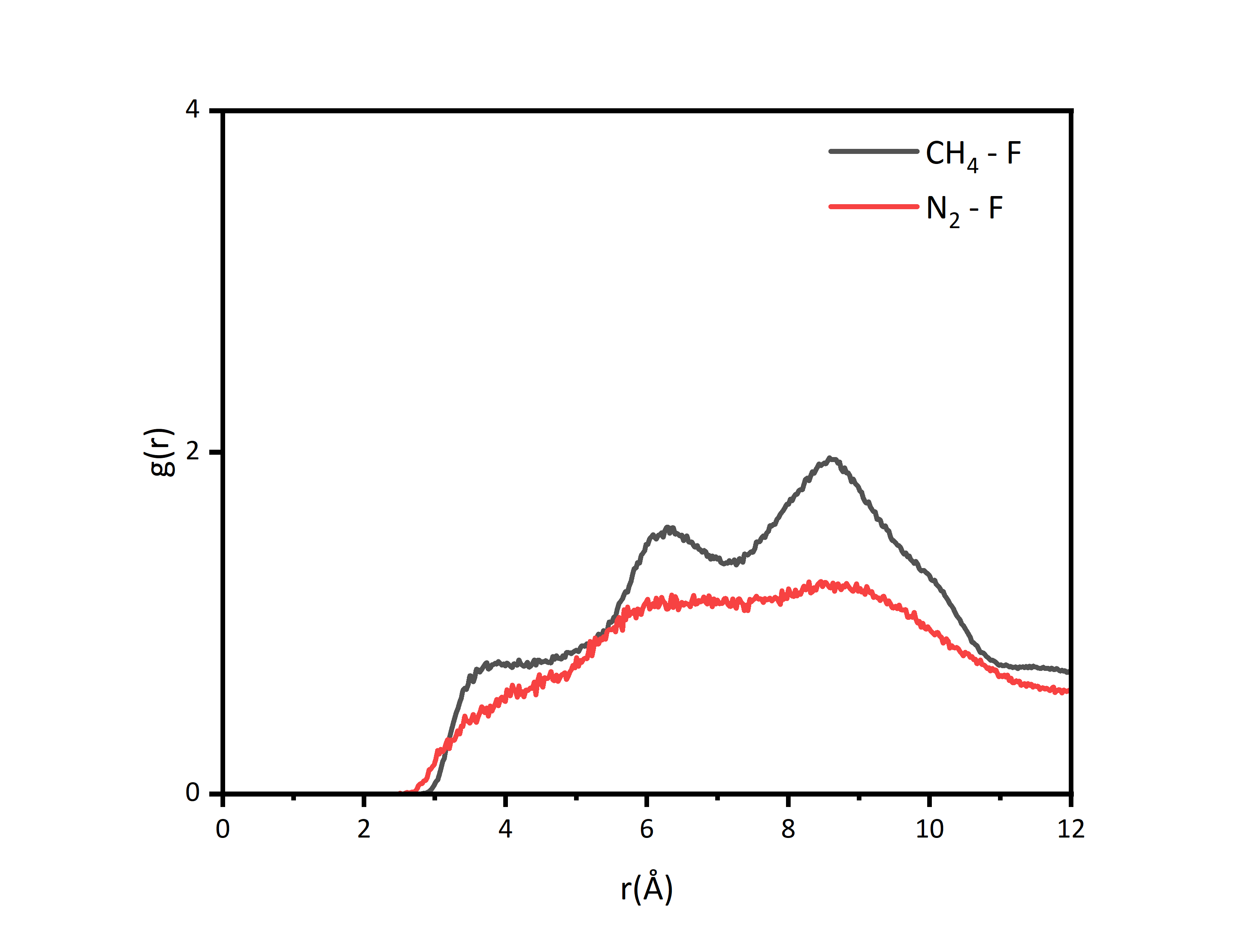

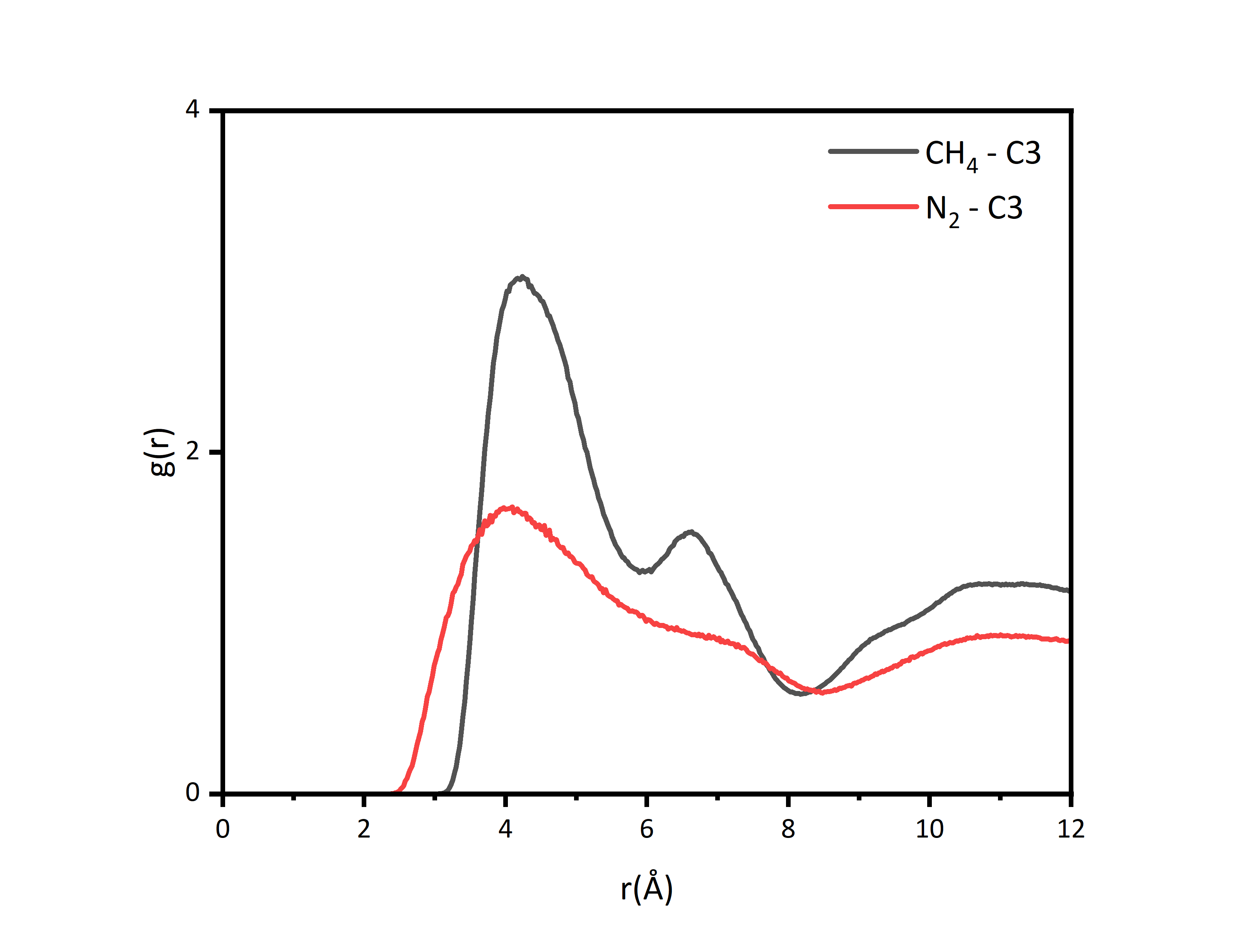

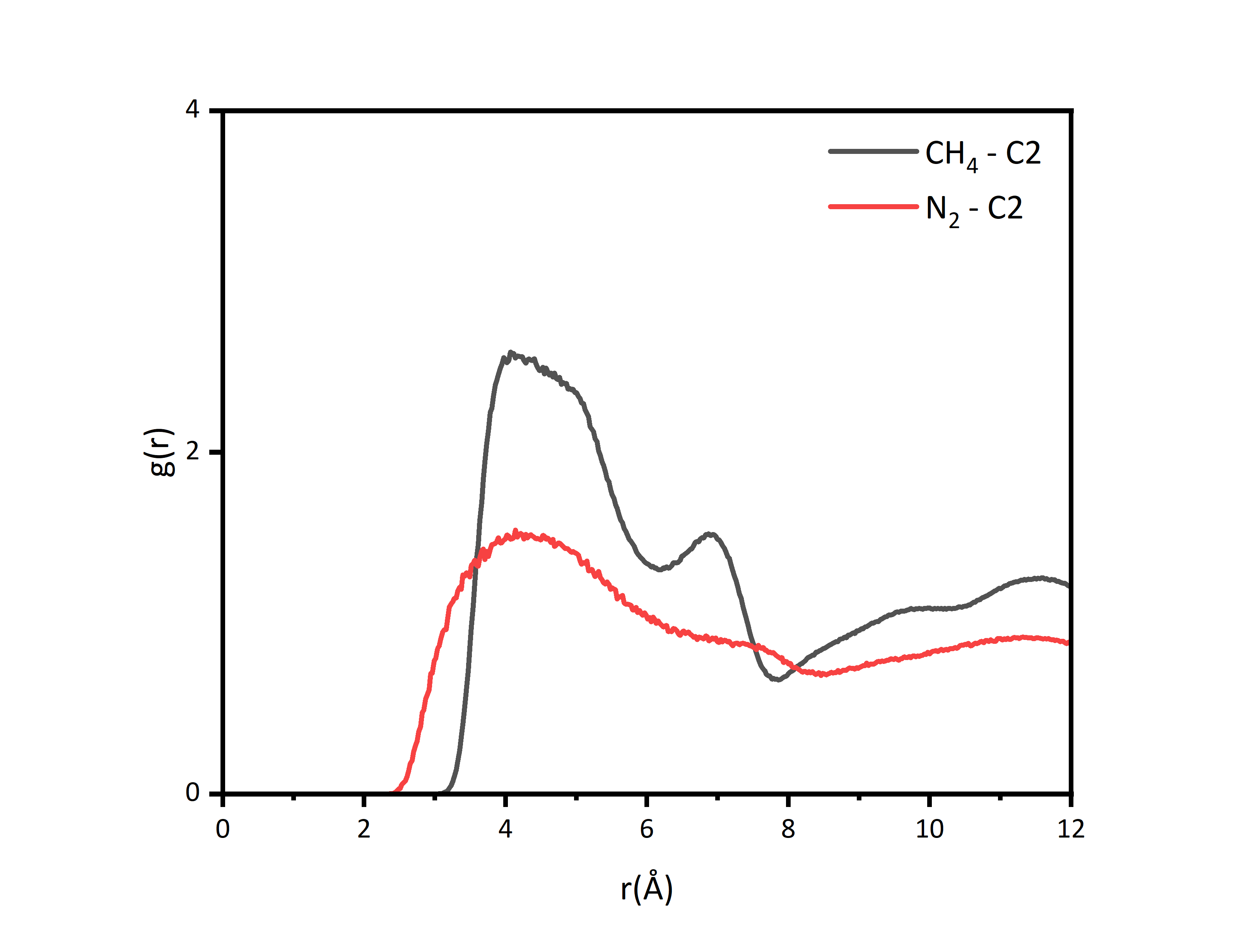

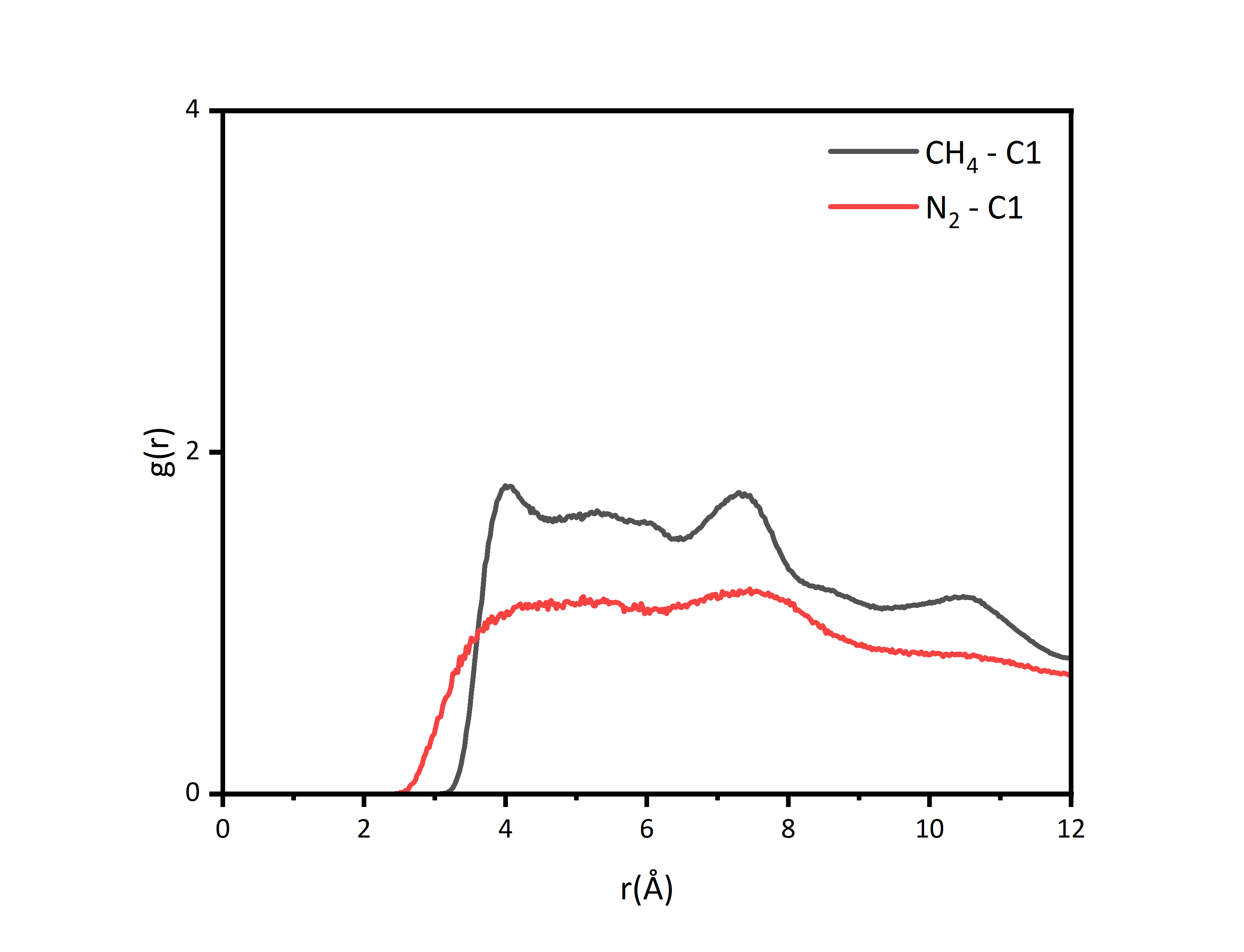

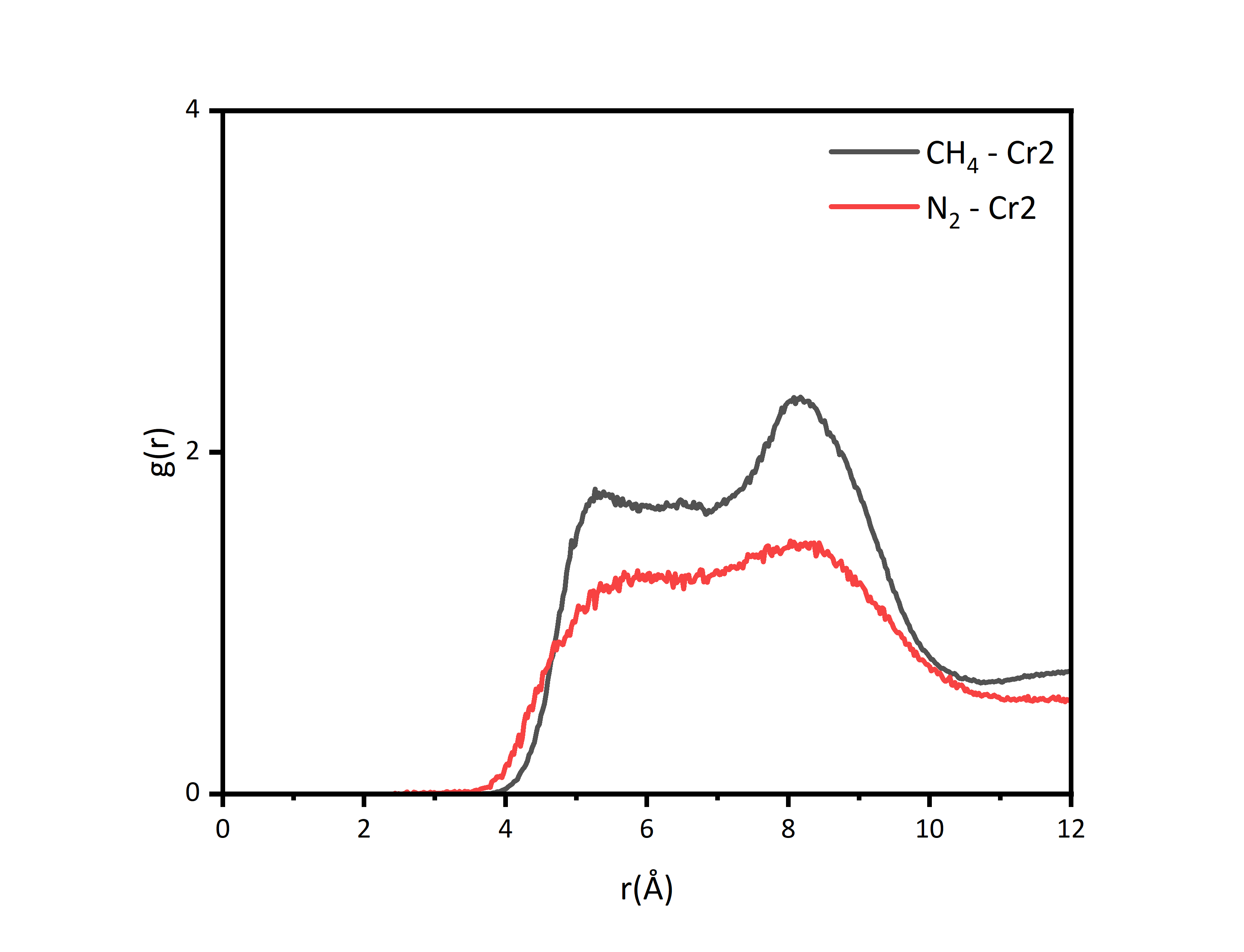

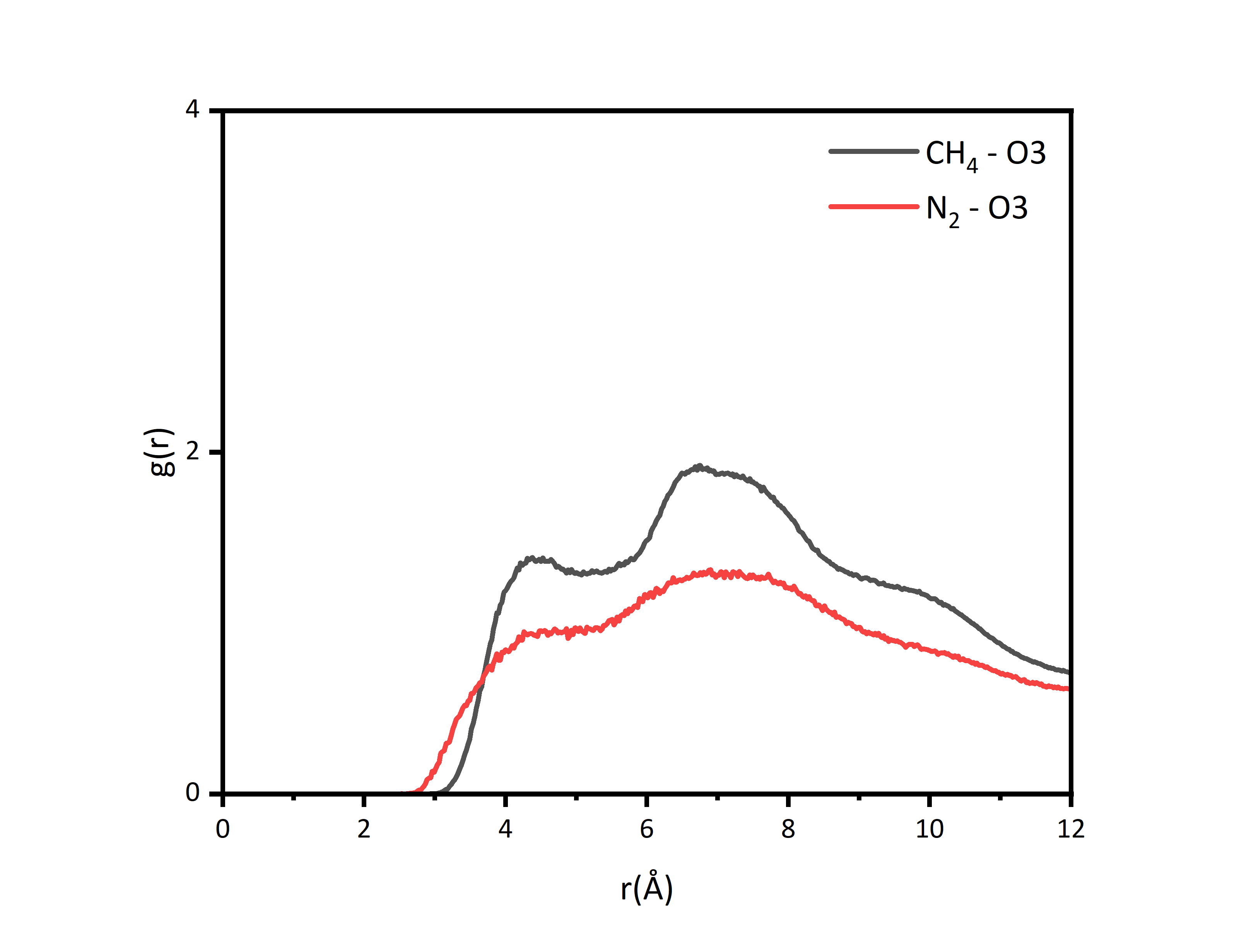

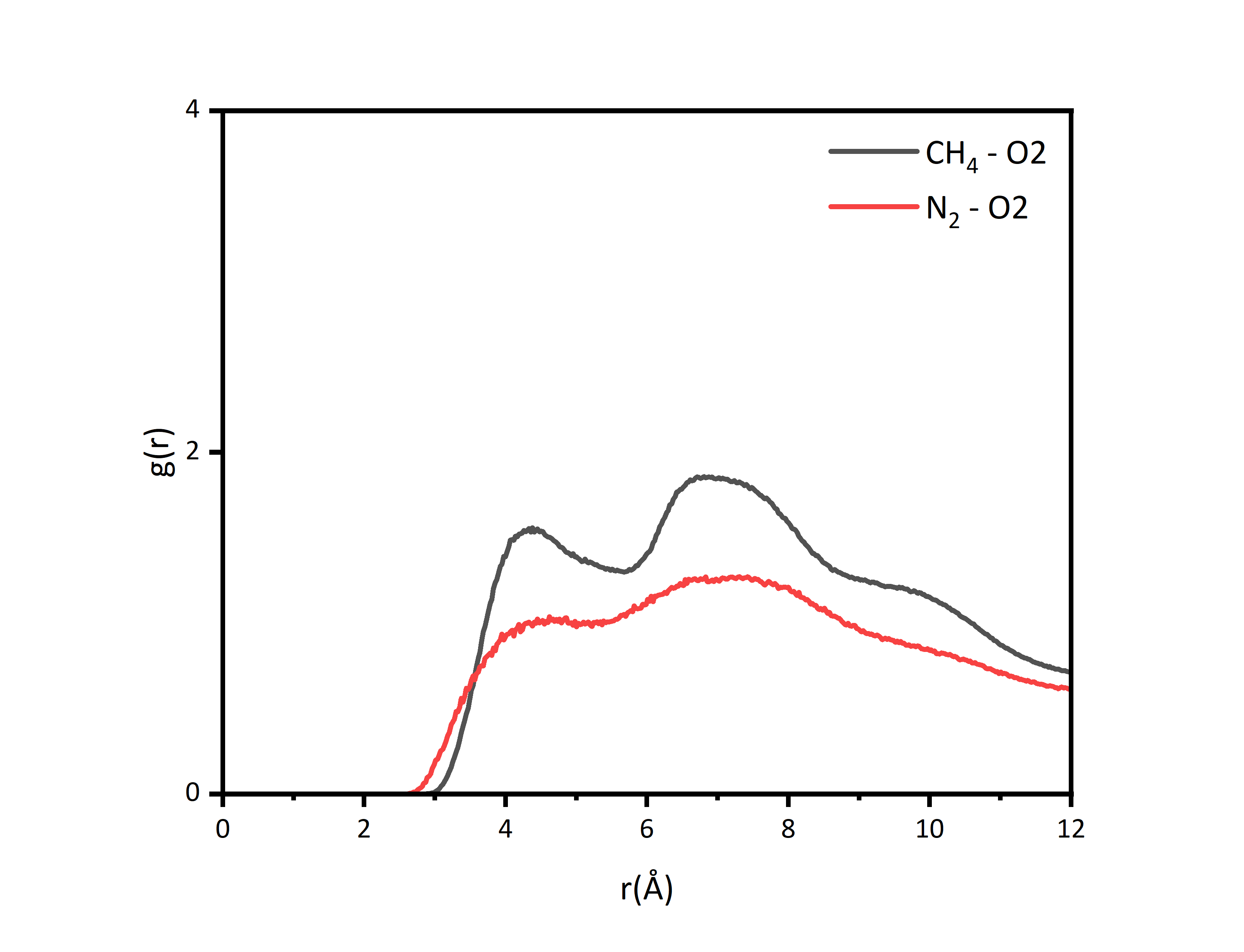

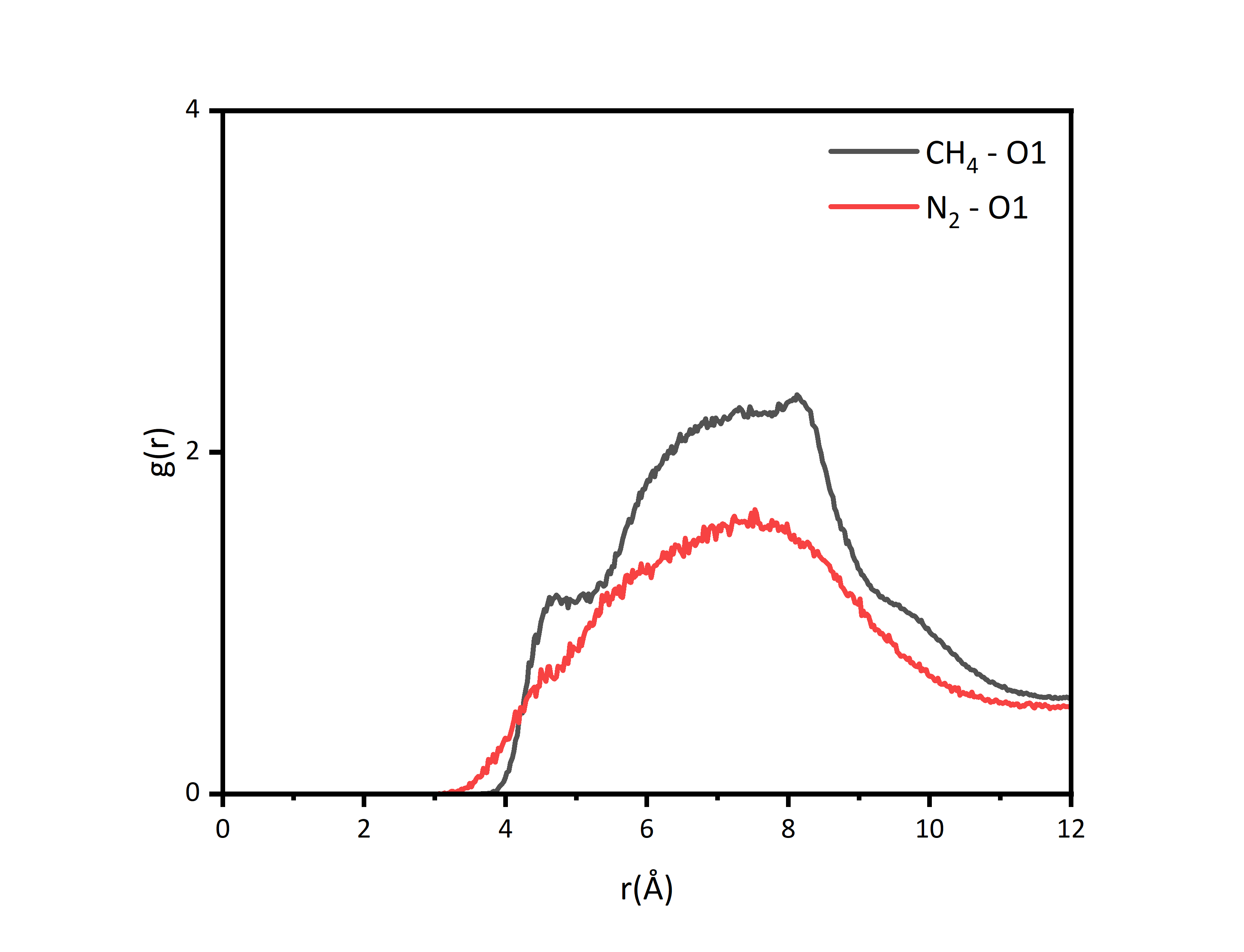

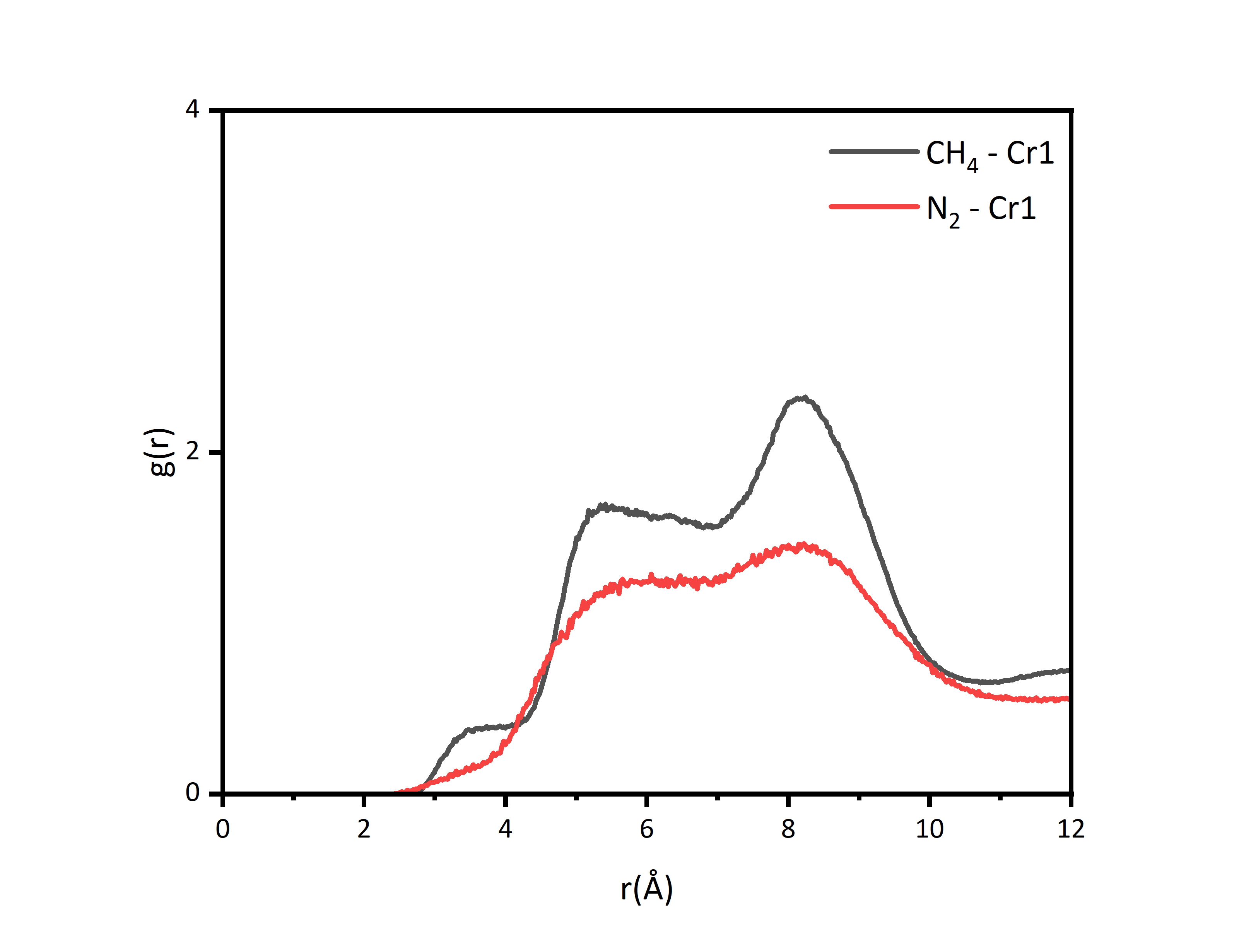


(d)

(c)

(b)

(a)

Figure S3. RDF Comparison N_2_ with CH_4_ around (a) Cr atoms; (b) O atoms; (c) C atoms; (d) F atoms in MIL-101.

(a)


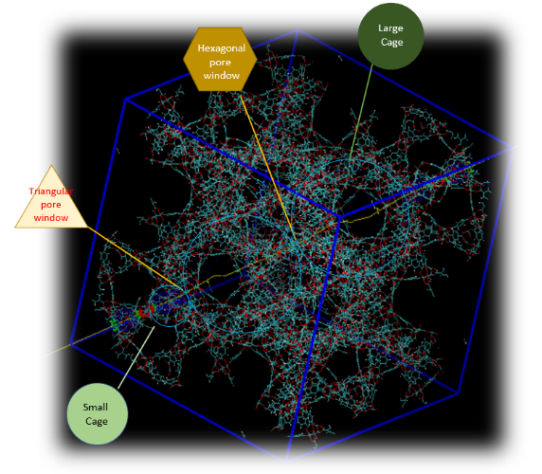

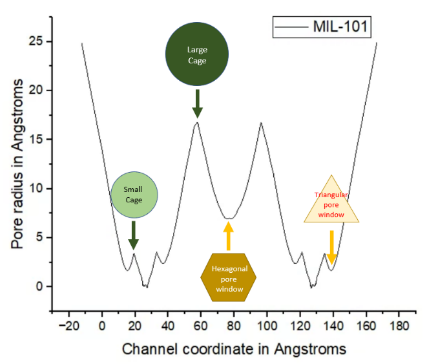

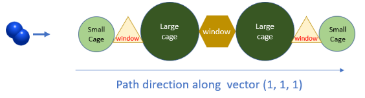


(b)


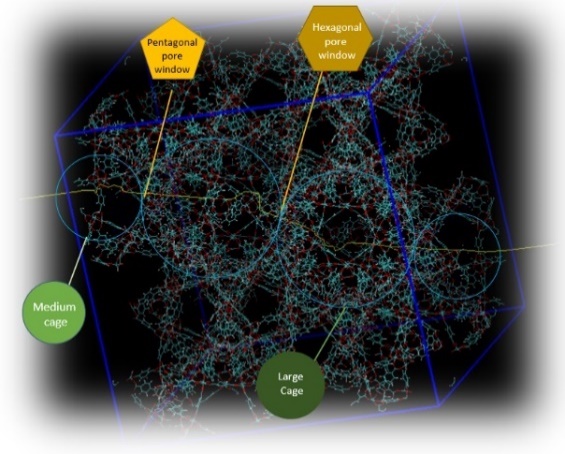

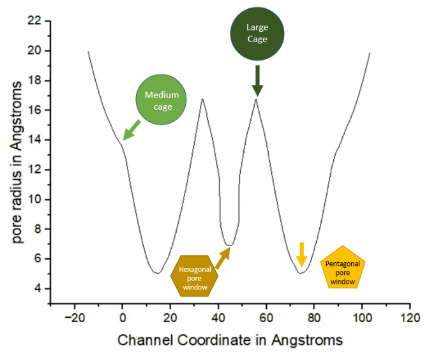

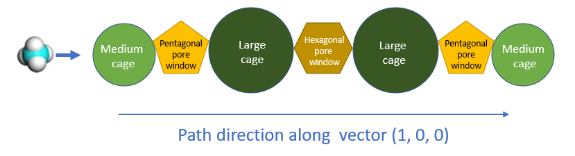


Figure S4. MIL-101 pore diameter through channel (a) along vector (1,1,1); (b) along vector (1, 0, 0).

(a)

(b)

Figure S5. (a) Effect of temperature on *D_s_*; (b) Arrhenius plots of the logarithm of self-diffusion coefficient as function of verse temperature for CH_4_ and N_2_.

Table S3. Force Field Parameters for MIL-101(Cr) Flexible Framework.


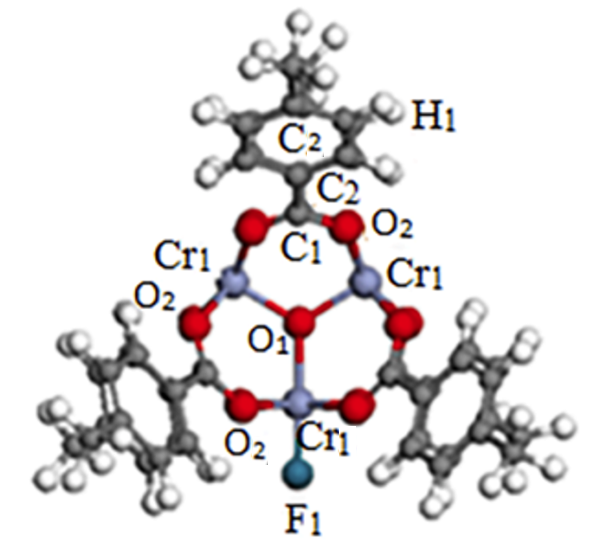


| Bond potential ^5^: $\boldsymbol{E=}\boldsymbol{K}_{\boldsymbol{b}}{\boldsymbol{(b-}\boldsymbol{b}_{\boldsymbol{0}}\boldsymbol{)}}^{\boldsymbol{2}}$ | | |
| --- | --- | --- |
| bond type | $\boldsymbol{K}_{\boldsymbol{b}}$ **(kcal∙mol^-1^A^-2^)** | $\boldsymbol{b}_{\boldsymbol{0}}$ **(Å)** |
| Cr1-O2 | 266.461876 | 1.918336 |
| Cr1-O1 | 285.091468 | 1.875606 |
| O2-C1 | 646.680887 | 1.311940 |
| C2-H1 | 357.440381 | 1.081418 |
| C2-C2 | 462.655054 | 1.379256 |
| C2-C1 | 391.669513 | 1.458000 |
| Cr1-F1 | 222.747130 | 1.853771 |

| Bending potential ^6^: $\boldsymbol{E=}\frac{\boldsymbol{2.0}}{\boldsymbol{n}^{\boldsymbol{2}}}\boldsymbol{C}\left[ \boldsymbol{1-B}\left( \boldsymbol{-1} \right)^{\boldsymbol{n}}\boldsymbol{cos}\left( \boldsymbol{n\theta} \right) \right]$ | | | |
| --- | --- | --- | --- |
| angle type | ***C* (kcal∙mol^-1^rad^-2^)** | ***B*** | ***n*** |
| O2-Cr1-O2 | 131.96075 | 1 | 4 |
| O2-Cr1-O1 | 136.40927 | 1 | 4 |
| Cr1-O2-C1 | 105.13339 | -1 | 3 |
| H1-C2-C2 | 57.289020 | -1 | 3 |
| C2-C2-C2 | 111.29751 | -1 | 3 |
| O2-C1-O2 | 187.13633 | -1 | 3 |
| O2-C1-C2 | 131.75571 | -1 | 3 |
| C1-C2-C2 | 102.18328 | -1 | 3 |
| O2-Cr1-F1 | 104.63644 | 1 | 4 |
| F1-Cr1-O1 | 108.37254 | 1 | 4 |
| Cr1-O1-Cr1 | 73.442750 | -1 | 3 |

| Dihedral potential ^7^: $\boldsymbol{E=K}\left[ \boldsymbol{1+dcos}\left( \boldsymbol{n\emptyset} \right) \right]$ | | | |
| --- | --- | --- | --- |
| type | ***K* (kcal∙mol^-1^)** | ***d*** | ***n*** |
| Cr1-O2-C1-O2 | 6.73711 | -1 | 2 |
| H1-C2-C2-H1 | 3.36856 | -1 | 2 |
| O2-C1-C2-C2 | 1.25000 | -1 | 2 |

| Improper potential ^8^: $\boldsymbol{E=K}\left[ \boldsymbol{C}_{\boldsymbol{0}}\boldsymbol{+}\boldsymbol{C}_{\boldsymbol{1}}\boldsymbol{cos}\left( \boldsymbol{\omega} \right)\boldsymbol{+}\boldsymbol{C}_{\boldsymbol{2}}\boldsymbol{cos}\left( \boldsymbol{2}\boldsymbol{\omega} \right) \right]$ | | | | |
| --- | --- | --- | --- | --- |
| type | ***K* (kcal∙mol^-1^rad^-2^)** | $\boldsymbol{C}_{\boldsymbol{0}}$ | $\boldsymbol{C}_{\boldsymbol{1}}$ | $\boldsymbol{C}_{\boldsymbol{2}}$ |
| H1-C2-C2-C1 | 2 | 1 | -1 | 0 |

Table S4. Self-diffusion coefficients of CH_4_ and N_2_ molecules in MIL-101.

| Gas Molecules  (Number) | Self-Diffusion Coefficient (m^2^/s) | |
| --- | --- | --- |
|  | CH_4_ ^α^ | N_2_ ^α^ |
| 100 | **5.4217E-09** (9.4448E-10) | **2.0106E-08** (1.0962E-09) |
| 200 | **6.1780E-09** (1.6990E-10) | **2.1941E-08** (1.0384E-09) |
| 400 | **1.0082E-08** (1.3012E-09) | **2.2747E-08** (8.4450E-10) |
| 600 | **1.2600E-08** (6.9105E-10) | **2.3245E-08** (5.8664E-10) |
| 800 | **1.5219E-08** (5.2838E-10) | **2.4821E-08** (6.9116E-10) |

***^α^***The values in parentheses represent the uncertainty values.

Table S5. Effect of temperature on self-diffusion coefficients of CH_4_ and N_2_ with 200 molecules.

| Temperature  (K) | Self-Diffusion Coefficient (m^2^/s) | |
| --- | --- | --- |
|  | CH_4_ ^α^ | N_2_ ^α^ |
| 263 | **4.3682E-09** (8.1217E-10) | **1.2684E-08** (8.9379E-10) |
| 273 | **5.5636E-09** (1.0628E-09) | **1.4345E-08** (1.0062E-09) |
| 283 | **6.3111E-09** (1.5845E-09) | **1.5188E-08** (1.2107E-09) |
| 300 | **7.2095E-09** (7.3811E-10) | **1.6152E-08** (1.3495E-09) |
| 310 | **8.4751E-09** (8.9086E-10) | **1.7788E-08** (1.3935E-09) |

***^α^***The values in parentheses represent the uncertainty values.

Table S6. Self-diffusion coefficients of CH_4_ and N_2_ when loading 200 CH_4_ molecules in MIL-101 at different loadings of N_2_ molecules at 298 K.

| N_2_ Molecules  (Number) | Self-Diffusion Coefficient (m^2^/s) | |
| --- | --- | --- |
|  | Mix CH_4_ ^α^ | Mix N_2_ ^α^ |
| 100 | **7.4211E-09** (8.7158E-10) | **1.5315E-08** (1.8131E-09) |
| 200 | **7.9893E-09** (1.8262E-09) | **1.7214E-08** (1.5805E-09) |
| 400 | **8.9448E-09** (2.5847E-10) | **2.0000E-08** (1.1141E-09) |
| 600 | **9.8696E-09** (6.6682E-10) | **2.0495E-08** (1.1776E-09) |
| 800 | **1.1076E-08** (8.6352E-10) | **2.1573E-08** (2.1219E-09) |

***^α^***The values in parentheses represent the uncertainty values.

Table S7. Self-diffusion coefficients of N_2_ when compared loading 0 CH_4_ molecules with 200 CH_4_ molecules at different loadings of N_2_ molecules at 298 K.

| N_2_ Molecules  (Number) | Self-Diffusion Coefficient (m^2^/s) | N_2_ Molecules  (Number) | Self-Diffusion Coefficient (m^2^/s) |
| --- | --- | --- | --- |
|  | Mix N_2_ ^α^ |  | Single N_2_ ^α^ |
| 100 | **1.5315E-08** (1.8131E-09) | **100** | **2.0106E-08** (1.0962E-09) |
| 200 | **1.7214E-08** (1.5805E-09) | **200** | **2.1941E-08** (1.0384E-09) |
| 400 | **2.0000E-08** (1.1141E-09) | **400** | **2.2747E-08** (8.4450E-10) |
| 600 | **2.0495E-08** (1.1776E-09) | **600** | **2.3245E-08** (5.8664E-10) |
| 800 | **2.1573E-08** (2.1219E-09) | **800** | **2.4821E-08** (6.9116E-10) |

***^α^***The values in parentheses represent the uncertainty values.

Table S8. The experimental and simulated values for gas selectivity (CH_4_/N_2_).

| Sim | | | | Ref ^9^ | |
| --- | --- | --- | --- | --- | --- |
| Pressure  (Bar) | **CH_4_**^α^  **(mmol/g)** | **N_2_**^α^  **(mmol/g)** | **Selectivity**  **(CH_4_/N_2_)** | **Pressure**  **(Bar)** | **Selectivity**  **(CH_4_/N_2_)** |
| 0.1 | **0.0187**  (0.0009) | **0.0009**  (0.0208) | **2.102**  (0.145) | **0.1** | 2.215 |
| 0.2 | **0.0352**  (0.0020) | **0.0020**  (0.0405) | **2.032**  (0.130) | **0.2** | 2.217 |
| 0.3 | **0.0518**  (0.0035) | **0.0035**  (0.0580) | **2.085**  (0.163) | **0.3** | 2.219 |
| 0.4 | **0.0677**  (0.0024) | **0.0024**  (0.0778) | **2.031**  (0.099) | **0.4** | 2.222 |
| 0.5 | **0.0837**  (0.0023) | **0.0023**  (0.0952) | **2.053**  (0.082) | **0.5** | 2.224 |
| 0.6 | **0.0983**  (0.0039) | **0.0039**  (0.1139) | **2.014**  (0.118) | **0.6** | 2.226 |
| 0.7 | **0.1155**  (0.0020) | **0.0020**  (0.1310) | **2.056**  (0.067) | **0.7** | 2.228 |
| 0.8 | **0.1272**  (0.0047) | **0.0047**  (0.1471) | **2.018**  (0.077) | **0.8** | 2.230 |
| 0.9 | **0.1431**  (0.0019) | **0.0019**  (0.1664) | **2.007**  (0.053) | **0.9** | 2.232 |

***^α^***The values in parentheses represent the uncertainty values.

Table S9. CH_4_  ^4^ and N_2_ ^10^ adsorption isotherms***^α^*** comparison of GCMC simulations and experimental measurements in MIL-101(Cr) at 298 K.

| Pressure (Bar) | CH_4_ - MIL101(Cr) | | | N_2_- MIL101(Cr) | | |
| --- | --- | --- | --- | --- | --- | --- |
|  | **CH_4_ Exp** | **CH_4_ Sim** | | **N_2_ Exp** | **N_2_ Sim** | |
|  | excess  adsorption  (mmole/g) | excess adsorption (mmole/g) | Number  (molecules  /UC) | excess  adsorption  (mmole/g) | excess adsorption (mmole/g) | Number  (molecules  /UC) |
| 1 | 0.4728 | **0.4587** (0.0065) | 86 | 0.2605 | **0.26832** (0.00529) | 50 |
| 10 | 2.4412 | **2.7724** (0.0057) | 455 | 1.0234 | **0.9439**  (0.0043) | 176 |
| 20 | 4.2032 | **4.5481** (0.0103) | 784 | 1.8394 | **2.0131**  (0.0137) | 376 |
| 30 | 5.7452 | **5.9980** (0.0070) | 1072 | 2.4754 | **2.4250**  (0.0031) | 452 |
| 40 | 7.0672 | **7.1879** (0.0253) | 1318 | 2.9314 | **3.0034**  (0.0024) | 560 |
| 50 | 8.1692 | **8.1025** (0.0290) | 1524 | 3.2074 | **3.1195**  (0.0077) | 582 |

***^α^***The values in parentheses represent the uncertainty values.

1. **Uncertainty Analysis**

**1.1 Standard Deviation Error Calculation**

Standard Deviation (SD) ^11^ is a fundamental statistical measure used to quantify the amount of variation or dispersion in a set of data values. In the context of uncertainty analysis, standard deviation provides a way to assess the precision of measurements and to estimate the uncertainty associated with those measurements, which are calculated from the equation shown below.

$$SD=\sqrt{\frac{1}{N-1}\sum_{i=1}^{N} \left( x_{i}-\bar{x} \right)^{2}}$$

Where $x_{i}$ refers to individual sample; $\bar{x}$ refers to the mean of samples; *N* refers to the sample size.

**1.2 Standard Deviation Error Propagation in Division**

When two measured values are divided and their errors are calculated using standard deviation, we can use the error propagation rule to compute the resulting error. The error propagation rule takes into account the standard deviations (i.e., errors) of the measured values and calculates the uncertainty of the result.

When we have two measured values *A* and *B* with their standard deviations (errors) $\sigma_{A}$ and $\sigma_{B}$ respectively. We intend to calculate the error of $Q=\frac{A}{B}$ .

According to the error propagation rule ^12^, the relative error of 𝑄 can be calculated using the following formula:

$$\left( \frac{\sigma_{Q}}{Q} \right)^{2}=\left( \frac{\sigma_{A}}{A} \right)^{2}+\left( \frac{\sigma_{B}}{B} \right)^{2}$$

Where $\sigma_{Q}$ is the standard deviation (error) of *Q*; $\sigma_{A}$ is the standard deviation (error) of *A*; $\sigma_{B}$ is the standard deviation (error) of *B*. Then $\sigma_{Q}$ can be calculated in this formula shown below:

$$\sigma_{Q}=Q\cdot\frac{\sigma_{Q}}{Q}$$

1. **Calculation of Isosteric Heat of Adsorption in GCMC**


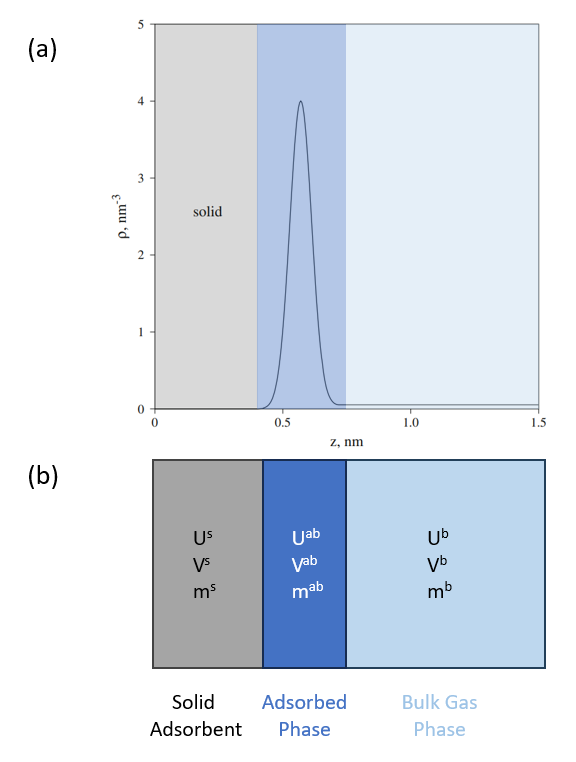


Figure S6. (a) The profile of gas density near the gas-solid interface is measured as a function of the distance z, which is taken perpendicular to the planar surface of the solid ^13^; (b) Schematic representation of the gas-solid interface ^14^.

The isosteric heat of adsorption is defined as the change in heat, *Q*, associated with the transfer of a single adsorbate molecule from the bulk gas phase to the adsorbed phase at constant temperature. Basic thermodynamic formulas indicate that *Q* can be viewed as the partial change in molar enthalpy during the adsorption process. However, in most instances, the enthalpy of the adsorbed phase is greater than that of the bulk phase. As a result, *Q_st_* is frequently interpreted as the negative partial change in molar enthalpy. The expression for calculating *Q_st_* can be formulated as follows ^15^:

$$\begin{aligned} Q_{st}=-\left( \frac{Q}{\partial N_{ad}} \right)_{T,P}=\left( \frac{\partial H_{b}}{\partial N_{b}} \right)_{T,P}-\left( \frac{\partial H_{ad}}{\partial N_{ad}} \right)_{T,P}\#\left( 2.1 \right) \end{aligned}$$

Under constant pressure, the change in enthalpy $\Delta H$ can be expressed by the following formula ^13^:

$$\begin{aligned} \Delta H= \Delta U+ P\Delta V \#\left( 2.2 \right) \end{aligned}$$

When the enthalpy $H$ is replaced by Eq. (2.2), Eq. (2.1) can be rewritten as ^15^:

$$\begin{aligned} Q_{st}=\left( \frac{\partial H_{b}}{\partial N_{b}} \right)_{T,P}-\left( \frac{\partial H_{ad}}{\partial N_{ad}} \right)_{T,P}=\left[ \left( \frac{\partial U_{b}}{\partial N_{b}} \right)_{T,P}+\left( \frac{\partial PV_{b}}{\partial N_{b}} \right)_{T,P} \right]-\left[ \left( \frac{\partial U_{ad}}{\partial N_{ad}} \right)_{T,P}+\left( \frac{\partial{PV}_{ad}}{\partial N_{ad}} \right)_{T,P} \right]\#\left( 2.3 \right) \end{aligned}$$

where the subscripts *ad* and *b* refer to the adsorbed and bulk phases, respectively. Here, *H*, *N*, *V*, and *U* represent enthalpy, particle number, volume, and total configurational energy, respectively.

In the case of a bulk phase, the perfect gas laws could be usually applied. So, *PV* is assumed to be equal to *nRT*, and the molar internal energy, $U_{b}$, for rigid molecules could be neglected as well ^16^. In the case of the adsorbed phase, the molecular volume of the adsorbed phase, $V_{ad}$, is neglected compared to that of the bulk phase, $V_{b}$, because $V_{b}\gg V_{ab}$ ^14^. Then, Eq. (2.3) can be rewritten as follow:

$$\begin{aligned} Q_{st}=RT-\left( \frac{\partial U_{ad}}{\partial N_{ad}} \right)_{T,P}\#\left( 2.4 \right) \end{aligned}$$

The partial derivative on the right-hand side of Eq. (2.4) can be computed directly using ensemble fluctuations:

$$\begin{aligned} \left( \frac{\partial U_{ad}}{\partial N_{ad}} \right)_{T,P}=\frac{f(U_{ad}, N_{ad})}{f(N_{ad}, N_{ad})}\#\left( 2.5 \right) \end{aligned}$$

where $f\left( X,Y \right)=\left\langle XY \right\rangle-\left\langle X \right\rangle\left\langle Y \right\rangle$ ^14^ represents the fluctuations of the X-Y pairs. So, Eq. (2.5) can be rewritten as follow ^15^:

$$\begin{aligned} Q_{st}=RT- \frac{\left\langle U_{ad}N_{ad} \right\rangle-\left\langle U_{ad} \right\rangle\left\langle N_{ad} \right\rangle}{\left\langle{N_{ad}}^{2} \right\rangle-\left\langle N_{ad} \right\rangle^{2}} \#\left( 2.6 \right) \end{aligned}$$

References

(1) Liang, Z.; Marshall, M.; Ng, C. H.; Chaffee, A. L. Comparison of Conventional and HF-Free-Synthesized MIL-101 for CO2 Adsorption Separation and Their Water Stabilities. *Energy & Fuels* **2013**, *27* (12), 7612-7618.

(2) Zhou, Z.; Mei, L.; Ma, C.; Xu, F.; Xiao, J.; Xia, Q.; Li, Z. A novel bimetallic MIL-101(Cr, Mg) with high CO2 adsorption capacity and CO2/N2 selectivity. *Chemical Engineering Science* **2016**, *147*, 109-117.

(3) Zhang, K.; Chen, Y.; Nalaparaju, A.; Jiang, J. Functionalized metal–organic framework MIL-101 for CO2 capture: multi-scale modeling from ab initio calculation and molecular simulation to breakthrough prediction. *CrystEngComm* **2013**, *15* (47), 10358-10366, 10.1039/C3CE41737A.

(4) Chowdhury, P.; Mekala, S.; Dreisbach, F.; Gumma, S. Adsorption of CO, CO2 and CH4 on Cu-BTC and MIL-101 metal organic frameworks: Effect of open metal sites and adsorbate polarity. *Microporous and Mesoporous Materials* **2012**, *152*, 246-252.

(5) LAMMPS. *Bond Styles: bond_style harmonic command*. Available Online: <https://docs.lammps.org/bond_harmonic.html>, 2024.

(6) LAMMPS. *Angle Styles: angle_style cosine/periodic command*. Available Online: <https://docs.lammps.org/angle_cosine_periodic.html>, 2024.

(7) LAMMPS. *Dihedral Styles: dihedral_style harmonic command*. Available Online: <https://docs.lammps.org/dihedral_harmonic.html> 2024.

(8) LAMMPS. *Improper Styles: improper_style fourier command*. Available Online: <https://docs.lammps.org/improper_fourier.html>, 2024.

(9) Singh, N.; Dalakoti, S.; Sharma, A.; Chauhan, R.; Murali, R. S.; Divekar, S.; Dasgupta, S.; Aarti. Shaping of MIL-53-Al and MIL-101 MOF for CO2/CH4, CO2/N2 and CH4/N2 separation. *Separation and Purification Technology* **2024**, *341*, 126820.

(10) Zhang, Z.; Wang, H.; Li, J.; Wei, W.; Sun, Y. Experimental Measurement of the Adsorption Equilibrium and Kinetics of CO2 in Chromium-Based Metal-Organic Framework MIL-101. *Adsorption Science & Technology* **2013**, *31* (10), 903-916.

(11) Farrance, I.; Frenkel, R. Uncertainty of Measurement: A Review of the Rules for Calculating Uncertainty Components through Functional Relationships. *The Clinical biochemist. Reviews* **2012**, *33* (2), 49-75.

(12) Fantner, G. A brief introduction to error analysis and propagation. *Script of” Laboratory of Nanoscale Biology”, EPFL. Feb* **2011**.

(13) Myers, A. L.; Monson, P. A. Physical adsorption of gases: the case for absolute adsorption as the basis for thermodynamic analysis. *Adsorption* **2014**, *20* (4), 591-622.

(14) Karavias, F.; Myers, A. L. Isosteric heats of multicomponent adsorption: thermodynamics and computer simulations. *Langmuir* **1991**, *7* (12), 3118-3126.

(15) Du, Z.; Nie, X.; Deng, S.; Zhao, L.; Li, S.; Zhang, Y.; Zhao, J. Comparative analysis of calculation method of adsorption isosteric heat: Case study of CO2 capture using MOFs. *Microporous and Mesoporous Materials* **2020**, *298*, 110053.

(16) Ungerer, P.; Tavitian, B.; Boutin, A. *Applications of Molecular Simulation in the Oil and Gas Industry: Monte Carlo Methods*; Editions Technip, 2005.
